# Clinical Efficacy of Enzalutamide vs Bicalutamide Combined With Androgen Deprivation Therapy in Men With Metastatic Hormone-Sensitive Prostate Cancer: A Randomized Clinical Trial

**DOI:** 10.1001/jamanetworkopen.2020.34633

**Published:** 2021-01-26

**Authors:** Ulka N. Vaishampayan, Lance K. Heilbrun, Paul Monk, Sheela Tejwani, Guru Sonpavde, Clara Hwang, Daryn Smith, Pallavi Jasti, Kimberlee Dobson, Brenda Dickow, Elisabeth I. Heath, Louie Semaan, Michael L. Cher, Joseph A. Fontana, Sreenivasa Chinni

**Affiliations:** 1 Department of Oncology, Karmanos Cancer Center, Wayne State University, Detroit, Michigan; 2 Department of Internal Medicine, University of Michigan, Ann Arbor; 3 Department of Internal Medicine, The Ohio State University, Columbus; 4 Department of Internal Medicine, Henry Ford Hospital, Detroit, Michigan; 5 Department of Internal Medicine, Dana Farber Cancer Institute, Boston, Massachusetts; 6 John D. Dingell Veterans Affairs Medical Center, Detroit, Michigan; 7 Department of Urology, Wayne State University, Detroit, Michigan

## Abstract

**Importance:**

Black patients have been underrepresented in prospective clinical trials of advanced prostate cancer. This study evaluated the efficacy of enzalutamide compared with bicalutamide, with planned subset analysis of Black patients with metastatic hormone-sensitive prostate cancer (mHSPC), which is a disease state responsive to androgen deprivation therapy (ADT).

**Objective:**

To compare the efficacy of enzalutamide vs bicalutamide in combination with ADT in men with mHSPC, with a subset analysis of Black patients.

**Design, Setting, and Participants:**

In this randomized clinical trial, a phase 2 screening design enabled a nondefinitive comparison of the primary outcome by treatment. Patients were stratified by race (Black or other) and bone pain (present or absent). Accrual of at least 30% Black patients was required. This multicenter trial was conducted at 4 centers in the US. Men with mHSPC with no history of seizures and adequate marrow, renal, and liver function were eligible. Data analysis was performed from February 2019 to March 2020.

**Interventions:**

Participants were randomized 1:1 to receive oral enzalutamide (160 mg daily) or bicalutamide (50 mg daily) in addition to ADT.

**Main Outcomes and Measures:**

The primary end point was the 7-month prostate-specific antigen (PSA) response (SMPR) rate, a previously accepted surrogate for overall survival (OS) outcome. Secondary end points included adverse reactions, time to PSA progression, and OS.

**Results:**

A total of 71 men (median [range] age, 65 [51-86] years) were enrolled; 29 (41%) were Black, 41 (58%) were White, and 1 (1%) was Asian. Thirty-six patients were randomized to receive enzalutamide, and 35 were randomized to receive bicalutamide. Twenty-six patients (37%) had bone pain and 37 patients (52%) had extensive disease. SMPR was achieved in 30 of 32 patients (94%; 95% CI, 80%-98%) taking enzalutamide and 17 of 26 patients (65%; 95% CI, 46%-81%) taking bicalutamide (*P* = .008) (difference, 29%; 95% CI, 5%-50%). Among Black patients, the SMPR was 93% (95% CI, 69%-99%) among those taking enzalutamide and 42% (95% CI, 19%-68%) among those taking bicalutamide (*P* = .009); among non-Black patients, the SMPR was 94% (95% CI, 74%-99%) among those taking enzalutamide and 86% (95% CI, 60%-96%) among those taking bicalutamide. The 12-month PSA response rates were 84% with enzalutamide and 34% with bicalutamide.

**Conclusions and Relevance:**

The findings of this randomized clinical trial comparing enzalutamide with bicalutamide suggest that enzalutamide is associated with improved outcomes compared with bicalutamide, in terms of the rate and duration of PSA response, in Black patients with mHSPC.

**Trial Registration:**

ClinicalTrials.gov Identifier: NCT02058706

## Key Points

**Question:**

Is enzalutamide combined with androgen deprivation therapy associated with better outcomes than treatment with bicalutamide in Black men with metastatic hormone-sensitive prostate cancer (mHSPC)?

**Findings:**

In a randomized clinical trial of 71 men with mHSPC, the 7-month prostate-specific antigen response rate was significantly improved with enzalutamide vs bicalutamide among Black patients but not among non-Black patients.

**Meaning:**

These findings suggest that treatment with enzalutamide is associated with improved outcomes vs bicalutamide in Black men with mHSPC, and incorporation of enzalutamide in the mHSPC treatment plan should be strongly considered.

## Teaser

This randomized clinical trial examines the efficacy of enzalutamide vs bicalutamide in combination with androgen deprivation therapy (ADT) in men with metastatic hormone-sensitive prostate cancer.

## Introduction

Androgen deprivation therapy (ADT) has been the backbone of systemic therapy in advanced prostate cancer. Standard therapy consists of testosterone suppression in combination with bicalutamide, which is a nonsteroidal androgen receptor antagonist. Enzalutamide is a second-generation androgen receptor inhibitor that shows enhanced cytotoxic effects in prostate cancer cells by blocking nuclear translocation and DNA binding.^1^ Compared with bicalutamide, enzalutamide has demonstrated superior progression-free survival in metastatic castrate-resistant prostate cancer (mCRPC).^2^ This study was designed to evaluate the clinical efficacy of enzalutamide vs bicalutamide in an earlier setting of metastatic hormone-sensitive prostate cancer (mHSPC).

Multiple ways of enhancing and optimizing therapy in hormone-sensitive disease have now been reported. The CHAARTED trial^3^ reported an overall survival (OS) advantage with the addition of docetaxel chemotherapy to ADT in patients with mHSPC. The STAMPEDE^4^ and LATITUDE^5^ studies proved the OS benefit of adding abiraterone and prednisone to ADT in patients with metastatic or high-risk prostate cancer. Recently, the TITAN^6^ trial affirmed the benefit of early apalutamide therapy. The addition of enzalutamide also reported proven efficacy in the ENZAMET^7^ and ARCHES^8^ trials. In summary, early intensification with the addition of an androgen receptor axis targeted (ARAT) agent, or docetaxel, demonstrated improved efficacy in mHSPC.

Although all these studies showed significant benefits, the populations enrolled were predominantly White. Black patients with advanced prostate cancer have specific nuances of presentation, prognosis, and therapy outcomes. Emerging data from population cohorts in the Veterans Affairs Health System revealed that Black men with prostate cancer have similar OS with the addition of docetaxel or abiraterone.^9^ The disparities in clinical outcomes, however, have not been prospectively evaluated in mHSPC. We conducted a randomized clinical trial evaluating clinical outcomes with early intensification of therapy in mHSPC and the impact on clinical outcomes in White and Black patients with prostate cancer.

Prostate cancer has a higher mortality rate among Black men compared with White men. A recent report^9^ reviewing the Veterans Affairs health database reported that Black men were younger and had higher prostate-specific antigen (PSA) levels but demonstrated no differences in 10-year prostate cancer–specific mortality. In metastatic prostate cancer, there appear to be differences in outcomes even when access to care is controlled, such as within a clinical trial patient population.^10^ The controversy is whether this disparity is explained by genetic or socioeconomic factors. Retrospective studies are confounded by multiple competing factors. Black patients are grossly underrepresented in prospective trials of advanced prostate cancer. The enrollment of Black patients was 10% in the CHAARTED^3^ trial and 12% in the SWOG 9346 trial.^11^ The ARCHES^8^ trial enrolled only 8 Black patients (1.4%) in each group, and no Black patients were enrolled in the TITAN^6^ and ENZAMET^7^ trials. Given the potential for racial disparity in prostate cancer outcomes and the underrepresentation of Black men in mHSPC clinical trials, the current study design required at least 30% enrollment of Black patients. The primary goal of this study was to compare the clinical outcomes with enzalutamide and bicalutamide in the mHSPC disease state, evaluating the differences in efficacy by race and exploring biomarkers associated with therapeutic resistance.

## Methods

### Study Design

The primary objective of this randomized clinical trial was to evaluate the 7-month PSA response (SMPR) rate in each group, because this has been reported to be a surrogate for OS in mHSPC.^11^ The secondary objectives were to evaluate the clinical safety, progression-free survival, time to PSA progression (TTPP), and OS, and to examine the associations of clinical outcomes with race and tissue biomarkers. This was a multicenter, open-label 1:1 randomized trial of enzalutamide at the starting dose of 160 mg (4 capsules of 40 mg each) orally daily vs bicalutamide 50 mg orally daily. Luteinizing hormone releasing hormone (LHRH) analogue therapy was administered in both groups. The study was approved by the Wayne State University institutional review board, and written informed consent was obtained from all patients before registration. Race was self-reported by patients and documented. This study follows the Consolidated Standards of Reporting Trials (CONSORT) reporting guideline. The trial protocol is provided in Supplement 1.

The primary objective of the study was to compare the rates of achieving PSA response at month 7 (SMPR) by treatment group with LHRH analogue therapy and enzalutamide vs LHRH analogue and bicalutamide. Secondary objectives included comparing the primary end point by race (Black vs non-Black), response rates, and adverse reactions in each group, and time to event end points (TTPP and OS) by treatment group and by race. The open-label study used a randomized phase 2 screening design, as described by Rubinstein et al.^12^ This enabled a nondefinitive comparison of the primary outcome by treatment. Patients were stratified by race (Black or other) and bone pain (present or absent) and were randomized within each of 4 strata. The randomization sequences consisted of random permuted blocks of varying sizes.

### Patient Selection

Eligible patients were aged 18 years or older, with histologically confirmed prostate adenocarcinoma with radiological evidence of metastases. Late induction was permitted within 3 months of starting LHRH analogue therapy or antiandrogen. A minimum PSA level of 4 ng/mL (to convert to micrograms per liter, multiply by 1) was required. Patients with a performance status score of 2 or less and life expectancy of 6 months or more were eligible. Patients were required to have adequate bone marrow, liver, and renal function. Patients with a history of neoadjuvant or adjuvant hormone therapy were eligible if they had received it for a duration of 24 months or less, consisting of ADT (single-agent LHRH or combination treatment with nonsteroidal androgen receptor inhibitors, excluding orchiectomy). Patients had to be not receiving ADT for at least 6 months before registration. No prior chemotherapy or ARAT agents were allowed. Concomitant radiation therapy was allowed for the palliation of severe pain or neuropathic compression. The study allowed a maximum of 2 dose reductions of enzalutamide to 120 mg and 80 mg orally daily, respectively. If grade 3 or 4 adverse reactions or grade 2 adverse reactions lasting for 7 days or longer were noted, then medication was held until the adverse reaction resolved to grade 1 or baseline. Dose reduction could be considered when resuming therapy. If an adverse reaction persisted after 2 dose reductions and optimal supportive care, then enzalutamide therapy had to be discontinued. Patients with history of seizures, risk factors for seizure, grade 3 or 4 congestive heart failure, or myocardial infarction were excluded. After the CHAARTED trial^3^ results, the study eligibility was modified to include patients with at least 1 of the following: low-volume disease (defined as no visceral metastases and <4 bone metastases), not candidates for docetaxel-based chemotherapy, or refused docetaxel chemotherapy.

### Tissue Biomarker Studies

#### Biopsy Tissue Procurement and Analysis

Metastatic biopsy collection was performed by a radiologist in the operating room. From each patient 1 to 3 cores were collected in a vial and flash frozen in liquid nitrogen, and another 1 to 2 cores were collected in 10% phosphate-buffered saline formaldehyde solution. Formalin-fixed bone biopsy tissues were decalcified in 10% EDTA solution before embedding in paraffin, whereas lymph node biopsy tissues were embedded after formalin fixation. Immunohistochemical analysis with pan-cytokeratin antibody was performed on paraffin-embedded tissue sections, and a pathologist assessed the tumor cell content in tissue sections. Tumor tissue biopsies positive for cytokeratin-expressing tumor cells were further processed for RNA extraction and quantitative polymerase chain reaction analysis.

#### Gene Expression Analysis

Frozen biopsy tissues were thawed and homogenized in a Precellys 24 tissue homogenizer (Bertin Instruments) in Trizol solution. RNA was extracted and quantitated with an Epoch microplate spectrophotometer from BioTek (ThermoFisher Scientific). One microgram of RNA was used for cDNA synthesis using a SuperScript First-strand synthesis kit (Invitrogen). Quantitative polymerase chain reaction analysis was performed with primers against *ERG*, *CXCR4*, and *AKR1C3* using methods described elsewhere (eFigure in Supplement 2).^13,14^ For absolute quantitation of gene expression in tumor tissue samples, first the standard curves were generated using plasmids harboring cDNA of genes in quantitative polymerase chain reaction (eFigure in Supplement 2). Using the standard curves, the copy numbers of gene expressed in tumor tissues were determined by extrapolating the cDNA copy number from tumor tissue circulating tumor values of genes of interest, as previously described.^15^

### Statistical Analysis

Statistical study design assumptions were (1) the proportion of patients achieving (and then sustaining) PSA level less than or equal to 4.0 ng/mL within 7 months of starting therapy (ie, the SMPR rate while taking bicalutamide) would be 50%; (2) the hypothesized SMPR rate while taking enzalutamide would be 75%; (3) α = .10 (1-sided); (4) power = 0.80; and (5) balanced 1:1 randomization. Those assumptions would require 41 patients per group (total of 82 patients), as calculated from the nQuery Advisor statistical software version 7.0 (Statsols). Accrual was halted after changes in standard of care. Hence, this report contains results for 71 accrued patients.

SMPR rates were compared using Fisher exact test, and 2-sided 95% CIs were calculated using the Wilson score method. TTPP and OS distributions were estimated with standard Kaplan-Meier methods. Median follow-up for TTPP, and separately for OS, was calculated using the reverse Kaplan-Meier method. Kaplan-Meier distributions were compared using stratified log-rank tests. The hazard ratio (HR) for PSA progression and its 2-sided 95% CI were derived from a Cox proportional hazards model. The HR for death (and its 2-sided 95% CI) was also derived from a Cox proportional hazards model. The proportional hazards assumption was checked by examining log(−log) plots and Epanechnikov smoothed hazard functions. Simultaneous comparison of 4-group (group by race) TTPP and of OS were followed by comparison of all 6 pairs of group and race subgroups. This created a multiple comparisons problem due to the resulting test multiplicity. To control the overall type 1 error rate (α) for the entire set of 6 comparisons, the Benjamini-Hochberg false discovery rate procedure, with a false discovery rate of 0.05, was used. Data analysis was performed using SAS statistical software version 9.4 (SAS Institute) from February 2019 to March 2020.

## Results

### Patient Characteristics and Adverse Reactions

**Figure 1.**
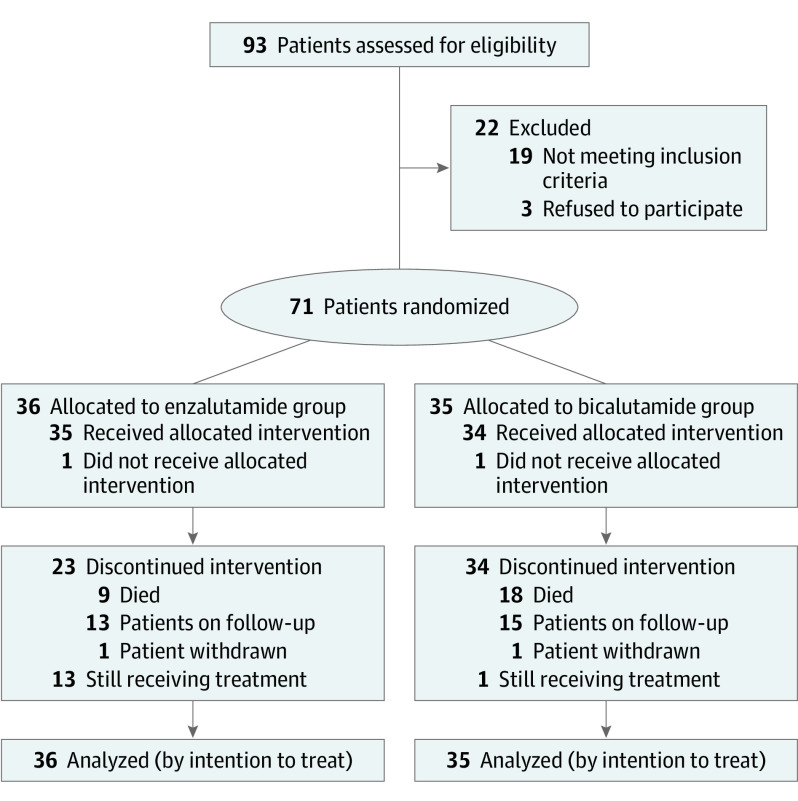
Study Recruitment Flowchart

A total of 71 men were enrolled; 29 (41%) were Black, 41 (58%) were White, and 1 (1%) was Asian (Figure 1). The median (range) age was 65 (51-86) years (Table 1). Thirty-six patients were randomized to receive enzalutamide, and 35 were randomized to receive bicalutamide. The study characteristics were well balanced between the 2 groups. The median (range) baseline PSA level was 56.3 ng/mL (4.2-10 431 ng/mL) in the enzalutamide group and 60 ng/mL (4.9-12 030 ng/mL) in the bicalutamide group. Twenty-six patients (37%) had bone pain, which is known to be associated with shorter survival outcome in patients with advanced prostate cancer. Forty-eight patients (68%) were late induction, 37 (52%) had extensive disease with 4 or more metastatic lesions, and 3 (4%) had visceral metastases. Predominant grade 3 adverse events in the enzalutamide group were hypertension in 3 patients and syncope in 2 patients (eTable in Supplement 2). One patient in the enzalutamide group had a seizure, and the medication was permanently discontinued. In the bicalutamide group, 1 patient had grade 3 hypertension and 2 patients had grade 3 fatigue. No grade 4 treatment-related adverse reactions were noted. No unexpected adverse reactions or treatment related mortality were noted.

**Table 1.** Patient Characteristics by Treatment Group

Characteristic	Patients, No. (%)		
	Total (N = 71)	Enzalutamide (n = 36)	Bicalutamide (n = 35)
Age, median (range), y	65 (51-86)	66 (54-86)	63 (51-84)
Black race	29 (41)	15 (42)	14 (40)
Bone pain			
Yes	26 (37)	14 (39)	12 (34)
No	45 (63)	22 (61)	23 (66)
Lung or liver metastases	3 (4)	1 (3)	2 (5)
Performance status score			
0	32 (45)	16 (44)	16 (45)
1	39 (55)	20 (56)	19 (54)
Bone metastases only	59 (83)	31 (86)	28 (80)
Extensive disease (≥4 lesions)	37 (52)	20 (56)	17 (49)
Oligometastatic disease (<4 lesions)	34 (48)	16 (44)	18 (51)
Measurable disease	34 (48)	17 (47)	17 (48)
Late induction	48 (68)	26 (72)	22 (63)

### Efficacy

#### Seven-Month PSA Response Rate

**Figure 2.**
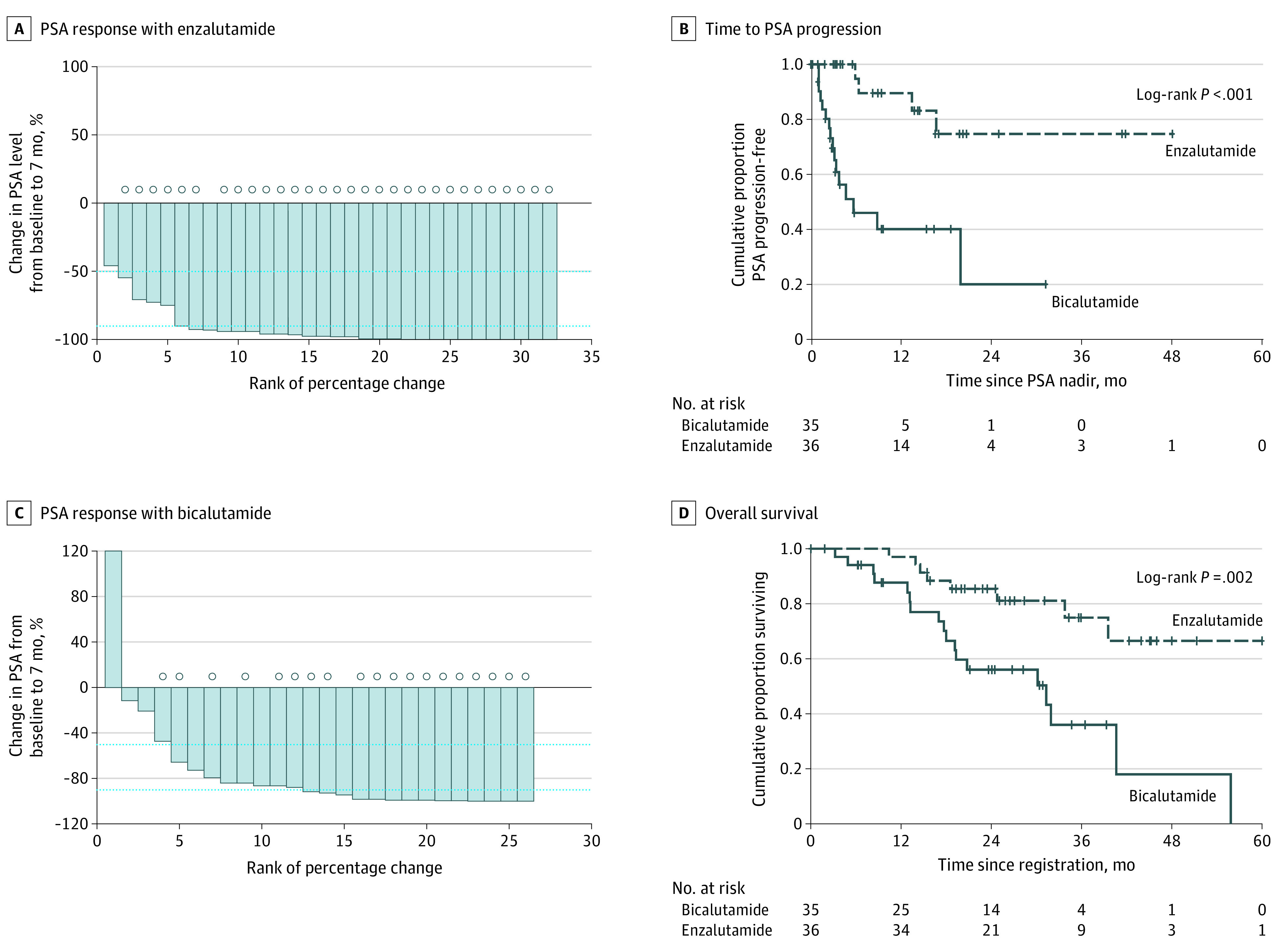
Prostate-Specific Antigen (PSA) Response, Time to PSA Progression, and Survival by Treatment Group Waterfall plots show PSA response with enzalutamide (A) and bicalutamide (C). In the waterfall plots, circles above the lines indicate nadir PSA level less than or equal to 4.0 ng/mL (to convert to micrograms per liter, multiply by 1). Panel B shows time to PSA progression. Panel D shows overall survival by treatment group. In B and D, vertical hash marks denote data censoring.

The SMPR end point is reported for 58 patients because 13 of the 71 patients enrolled did not have a month 7 PSA value. However, by the intention to treat principle, all 71 patients randomized are included in the analysis of clinical end points such as TTPP and OS. Waterfall plots in Figure 2A and 2B show PSA decreases for all patients. All 71 patients are included in the Kaplan-Meier graphs of TTPP and OS by therapy group (36 taking enzalutamide and 35 taking bicalutamide), and in the Kaplan-Meier graphs of TTPP and OS by race (42 non-Black and 29 Black). SMPR was achieved in 30 of 32 patients (94%; 95% CI, 80%-98%) taking enzalutamide and in 17 of 26 patients (65%; 95% CI, 46%-81%) taking bicalutamide (*P* = .008) (difference, 29%; 95% CI, 5%-50%) (Table 2). The Black patients had an increased likelihood of benefit with enzalutamide compared with bicalutamide, with SMPRs of 13 of 14 patients (93%; 95% CI, 69%-99%) and 5 of 12 patients (42%; 95% CI, 19%-68%), respectively (*P* = .009). The non-Black patients had comparable SMPR rates of 17 of 18 patients (94%; 95% CI, 74%-99%) taking enzalutamide and 12 of 14 patients (86%; 95% CI, 60%-96%) taking bicalutamide. The 12-month PSA response rates were 84% with enzalutamide and 34% with bicalutamide.

**Table 2.** The Primary End Point of SMPR Rate With Baseline Patient and Disease Characteristics Subgroups and Tissue Biomarker Data

Characteristic	Total (N = 71)^a^		Enzalutamide (n = 36)^b^		Bicalutamide (n = 35)^c^	
	Patients, No. (%)	SMPR rate, patients, No. (%)	Patients, No. (%)	SMPR rate, patients, No. (%)	Patients, No. (%)	SMPR rate, patients, No. (%)
Age, y						
<70	34 (59)	27 (79)	19 (59)	18 (95)	15 (58)	9 (60)
≥70	24 (41)	20 (83)	13 (41)	12 (92)	11 (42)	8 (73)
Race						
Black	26 (43)	18 (69)	14 (44)	13 (93)	12 (46)	5 (42)
Non-Black	32 (57)	29 (91)	18 (56)	17 (94)	14 (54)	12 (86)
Bone pain						
Yes	20 (34)	13 (65)	11 (34)	9 (82)	9 (35)	4 (44)
No	38 (66)	34 (89)	21 (66)	21 (100)	17 (65)	13 (76)
Gleason score						
≥8	40 (73)	32 (80)	23 (77)	21 (91)	17 (68)	11 (65)
<8	15 (27)	12 (80)	7 (23)	7 (100)	8 (26)	5 (63)
Sites of metastases						
Visceral	3 (5)	3 (100)	1 (3)	1 (100)	2 (8)	2 (100)
Lymph node	22 (38)	18 (82)	11 (34)	11 (100)	11 (42)	7 (64)
Bone only	25 (43)	19 (76)	17 (53)	15 (88)	8 (31)	4 (50)
Other	8 (13)	7 (88)	3 (9)	3 (100)	5 (19)	4 (80)
Performance status score						
0	27 (47)	25 (93)	15 (47)	15 (100)	12 (46)	10 (83)
1	31 (53)	22 (71)	17 (53)	15 (88)	14 (54)	7 (50)
*ERG* level						
Less than or equal to the median	22 (49)	19 (86)	10 (48)	10 (100)	12 (50)	9 (75)
Greater than the median	23 (51)	18 (78)	11 (52)	11 (100)	12 (50)	7 (58)
*CXCR4* level						
Less than or equal to the median	22 (49)	19 (83)	10 (48)	10 (100)	12 (50)	9 (75)
Greater than the median	23 (51)	18 (78)	11 (52)	11 (100)	12 (50)	7 (58)
*AKR1C3* level						
Less than or equal to the median	22 (49)	19 (86)	10 (48)	10 (100)	12 (50)	9 (75)
Greater than the median	23 (51)	18 (78)	11 (52)	11 (100)	12 (50)	7 (58)

Abbreviations: PSA, prostate specific antigen; SMPR, 7 month PSA response rate.

^a^ Not all patients with a 7-month PSA value had biomarker data. Also, not all patients with biomarker data had a 7-month PSA value. Thus, only 58 patients had SMPR data available. For all 71 patients (median [range] age, 66 [51-86] years), the SMPR rate was 81.0% (95% CI, 69.2%-89.1%).

^b^ For the enzalutamide group (median [range] age, 67 [54-86] years), the SMPR rate was 93.8% (95% CI, 79.9%-98.3%).

^c^ For the bicalutamide group (median [range] age, 64 [51-84] years), the SMPR rate was 65.4% (95% CI, 46.2%-80.6%).

#### Time to Event End Points

**Figure 3.**
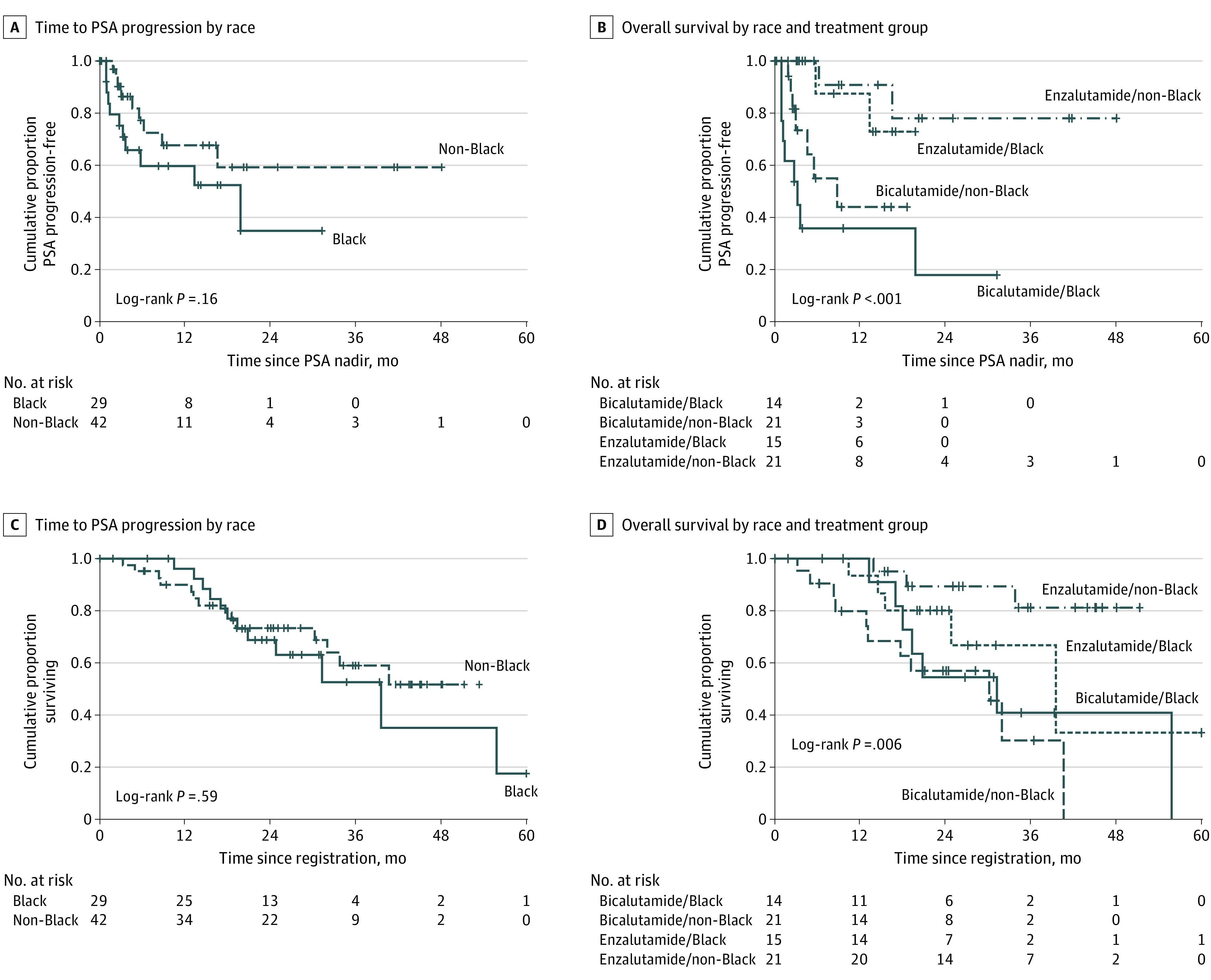
Prostate-Specific Antigen (PSA) Response, Time to PSA Progression, and Survival by Treatment Group and Race Graphs show time to PSA progression by race (A), overall survival by race and treatment group (B), time to PSA progression by race (C), and overall survival by race and treatment group (D). Vertical hash marks denote data censoring. To convert to PSA level micrograms per liter, multiply by 1.

Of the 71 patients, as of this writing, only 20 have had PSA progression, for an event proportion of 28%. Twenty-six (37%) deaths have occurred. This indicates immature TTPP and OS data, and follow-up is ongoing. The median (range) follow-up for TTPP was 8.6 (0.2-48.1) months. The median (range) follow-up for OS was 39.0 (0.4-60.7) months. Enzalutamide therapy significantly improved TTPP (HR, 0.15; 95% CI, 0.05-0.47; *P* < .001) and OS (HR, 0.31; 95% CI, 0.13-0.74; *P* = .002) (Figure 2C and 2D). TTPP did not differ significantly by race (HR for Black patients, 1.85; 95% CI, 0.77-4.47; *P* = .16) (Figure 3A). Also, OS did not differ significantly by race (HR for Black patients, 1.24; 95% CI, 0.56-2.72; *P* = .60) (Figure 3B). TTPP did not differ significantly by baseline bone pain status (*P* = .43; log-rank χ^2^_1_ = 0.63).

The overall difference in TTPP across the 4 groups of group and race combinations appeared to be clinically meaningful, and was significant (*P* < .001; log-rank χ^2^_3_ = 17.78) (Figure 3C). That finding does not identify which subgroups differed from each other. Comparison of all 6 pairs of subgroups was then required, while also controlling for the resulting test multiplicity. That was done using the Benjamini-Hochberg false discovery rate procedure, which revealed that only 2 subgroup comparisons were still significant using a false discovery rate of 0.05: the enzalutamide-treated non-Black patients had significantly longer TTPP than did the bicalutamide-treated non-Black patients (Benjamini-Hochberg adjusted *P* = .002; log-rank χ^2^_1_ = 13.21), and the enzalutamide-treated Black patients had significantly longer TTPP than the bicalutamide-treated Black patients (Benjamini-Hochberg adjusted *P* = .005; log-rank χ^2^_1_ = 9.79).

Somewhat similar subgroup comparison results were observed for OS (Figure 3D). Among non-Black patients, OS was significantly longer in the enzalutamide group than in the bicalutamide group (Benjamini-Hochberg adjusted *P* = .004; log-rank χ^2^_1_ = 11.62). Also, OS was significantly longer for enzalutamide-treated non-Black patients than for bicalutamide-treated Black patients (adjusted *P* = .04; log-rank χ^2^_1_ = 6.30).

#### Biomarkers

Of the 71 enrolled patients, 53 biopsy samples (75%) had tumor tissue available. Of these, the biomarkers were measurable in 45 samples. *TMPRSS-ERG* fusion gene, *CXCR4*, and androgen biosynthetic enzyme *AKR1C3* levels were determined in metastatic biopsies (eFigure in Supplement 2). Patients in the bicalutamide group with a low copy number of *ERG*, *CXCR4*, or *AKR1C3* had a higher SMPR (9 of 12 patients [75%]) compared with those with a high copy number (7 of 12 patients [58%]) (Table 2). However, no differences in SMPR were noted by race in the enzalutamide group, because both groups had almost 100% incidence rates. Black patients with high levels of *ERG*, *CXCR4*, or *AKR1C3* demonstrated a numerically lower likelihood of achieving SMPR compared with non-Black patients (67% vs 92%).

## Discussion

To our knowledge, this is the only randomized clinical trial comparing bicalutamide with enzalutamide in a Black patient population. The study results reveal that Black patients treated with bicalutamide had a lower likelihood of biochemical response than the non-Black patients (42% vs 86%). The SMPR in Black and non-Black patients taking enzalutamide therapy were comparable (93% vs 94%). Enzalutamide demonstrated a greater magnitude of improvement over bicalutamide, in the rate and duration of PSA response in Black patients. ADT plus bicalutamide is inadequate therapy in all cases, but especially for Black patients. The addition of enzalutamide therapy resulted in outcomes for Black patients comparable to those for non-Black patients. Retrospective studies^16,17,18^ have demonstrated racial differences in the efficacy of ARAT therapies. A matched case-control study^16^ led by Duke investigators confirmed that abiraterone and prednisone therapy improved PSA response rates and progression-free survival in Black patients compared with White patients with mCRPC. Another retrospective study^17^ showed that the addition of abiraterone and prednisone, or enzalutamide, resulted in Black patients having improved OS (HR, 0.887). Multivariable retrospective analysis^18^ of registry data evaluating sipuleucel-T therapy in mCRPC revealed that Black race was significantly associated with longer OS outcomes. The current study confirms that the racial differences in bicalutamide efficacy are overcome by using contemporary ARAT such as enzalutamide in advanced prostate cancer.

We explored the underlying molecular mechanisms that may be potential reasons for the differences in responses. *TMPRSS-ERG* fusion gene expression is highly prevalent in patients with prostate cancer.^19^ We have previously shown that a prometastatic gene *CXCR4*^20^ and an androgen biosynthetic enzyme *AKR1C3*^21^ are downstream-regulated genes of *TMPRSS2-ERG* fusions in prostate cancer cells. High baseline *ERG* levels and changes in androgen metabolism enzymes and resistance pathways such as *CXCR4* maybe potential molecular basis for the noted differences. Further investigations exploring molecular biomarkers to help guide therapy are highly recommended.

### Limitations and Strengths

Study limitations included the need to administratively close the study to accrual slightly short of the target sample size. That decision was essential after a phase 3 trial^5^ demonstrated that abiraterone and prednisone improved OS in mHSPC. We could no longer ethically randomize patients to bicalutamide. The impact of that decision on our study’s results and conclusions is minimal, and any small loss of statistical power is unimportant because a significant difference in favor of the enzalutamide group was still observed. A second study limitation is the immature time to event data, with event rates of 28% for TTPP and 37% for death.

Study strengths include the prospective nature of our study, the stratification by race, and the accrual of 41% Black patients. Cumulatively, these factors make the study conclusions meaningful for clinical application.

## Conclusions

In this study, Black men with mHSPC had a decreased response rate with ADT and bicalutamide. The incorporation of enzalutamide in mHSPC therapy overcame the disparity in biochemical response rate and OS outcomes between Black and non-Black patients. The regimen was well tolerated, and the favorable risk benefit profile makes it crucial to strongly consider the addition of enzalutamide to ADT in Black patients with mHSPC.

## Notes

Supplement 1. **Trial Protocol**

Supplement 2. **eTable.** Grade 3 Toxicities by Treatment Arm That Were Clinically Relevant and Deemed to Be Probably or Definitely Related to Therapy (N = 69) **eFigure.** Standard Curves for Biomarkers

Supplement 3. **Data Sharing Statement**
