# Supplementary material for: Clinical Efficacy of Enzalutamide vs Bicalutamide Combined With Androgen Deprivation Therapy in Men With Metastatic Hormone-Sensitive Prostate Cancer: A Randomized Clinical Trial
Source: JAMA Netw Open. 2021 Jan 26;4(1):e2034633. doi: 10.1001/jamanetworkopen.2020.34633 (PMC7838941; doi:10.1001/jamanetworkopen.2020.34633)
Supplement: Supplement 1. — Trial Protocol [file jamanetwopen-e2034633-s001.pdf]

Karmanos Cancer Institute (KCI) Protocol # 2013-083
Version: July 1, 2013, Sept 25 2013, Jan 16, 2014, May15, 2014, June 27, 2014, June 5, 2015, February 17, 2017, July 1, 2018, Feb 20, 2020, March 20,2020

Title: **Randomized Phase II screening trial of enzalutamide/MDV-3100 and LHRH analogue vs combined androgen deprivation (LHRH analogue + bicalutamide) in metastatic hormone sensitive prostate cancer**

**Principal Investigator:**	Elisabeth Heath, MD.<br>4 Hudson-Webber Cancer Research Center<br>4100 John R Road<br>Detroit MI 48201.<br>Tel # 313-576-8735<br>Fax # 313-576-8767<br>E mail: heathe@karmanos.org
**Co-Investigators:**	Frank Cackowski, MD<br>4100 John R Road<br>Detroit, MI 48201<br>Tel# 313-576-8321<br>Fax # 313-576- 8766<br>Email: cackowskif@karmanos.org
**Biostatistician :**	**Lance Heilbrun, Ph.D**.<br>Biostatistics Core<br>Mid Med Research Building, 3rd Floor<br>87 East Canfield<br>Detroit MI 48201.<br>Tel# 313-576-8652<br>E mail: heilbrun@karmanos.org
**Study Coordinator:**	**Kimberlee Dobson**<br>Clinical Trials Office<br>Mid Med Research Building, 5th Floor<br>87 East Canfield<br>Detroit MI 48201<br>Tel # **313-576-9837**<br>E mail: dobsonk@karmanos.org
**Laboratory Co-Investigator:**	**Sreenivasa R. Chinni, Ph.D.**<br>Departments of Urology, Oncology and Pathology, KCI,<br>Wayne State University School of Medicine

9245 Scott Hall,
540 E Canfield Ave Detroit, MI 48201.
Tel # 313-577-1833
E mail: schinni@med.wayne.edu

Pharmacodynamics/ Correlatives

Julie Boerner PhD
Wayne State University/
Barbara Ann Karmanos Cancer Institute
Hudson-Webber Cancer Research Center,
Correlative Sciences Lab Rm 808
4100 John R. Street
Detroit, Michigan 48201
Tel.: 313-576-8351 (lab)
Email: boernerj@med.wayne.edu

Karri Stark
Wayne State University/
Barbara Ann Karmanos Cancer Institute
Hudson-Webber Cancer Research Center,
Correlative Sciences Lab Rm 808
4100 John R. Street
Detroit, Michigan 48201
Tel.: 313-576-8248 (lab)
Email: starkka@karmanos.org

Susan Land PhD
Applied Genomics Technology Center
CS Mott Center Bldg.
275 East Hancock St.
Detroit MI 48201
313-577-9605 (office phone)
313-577-0024 (lab phone)
E mail: Sland@med.Wayne.edu

Grants and Contracts:

Mary Jo O'Loughlin
Karmanos Cancer Institute
4100 John R MM00RA
Detroit, MI 48201
Tel # 313-578-4405
Fax # 313-831-4039
E mail: oloughli@karmanos.org

Participating Sites:

Henry Ford Hospital
PI- Sheila Tejwani M.D.
2799 W Grand Blvd
Detroit, MI 48202
(313) 916-2494 (Office)
(313) 876-2494 (Fax)
E mail stejwan1@hfhs.org

Ohio State University
PI- Paul Monk MD
320 W 10th Ave Suite A433
Columbus, OH 43210
Phone: (614) 366-7126
Fax: (614) 293-7525
E mail paul.monk@osumc.edu

The Cancer Institute of New Jersey
PI- Mark Stein MD
The Cancer Institute of New Jersey,
195 Little Albany Street,
New Brunswick, NJ 0890
Phone: (732) 235-6031
Fax: (732) 235-7493
Email: steinmn@umdnj.edu

The University of Alabama at Birmingham
PI- Dr Laxminarayanan NandagopalAssistant Professor of Medicine
UAB Comprehensive Cancer center
1720 2nd Ave. South
Birmingham, AL 35294-3280
Phone: 205-975-5715
Fax: 205-934-9573
Email: lnandagopal@uabmc.edu

Mount Sinai School of Medicine
PI- William K. Oh, M.D.
Chief, Division of Hematology and Medical Oncology Professor of Medicine and Urology Ezra M. Greenspan, MD Professor in Clinical Cancer Therapeutics Icahn School of Medicine at Mount Sinai Associate Director of Clinical Research The Tisch Cancer Institute
New York, N.Y. 10029
Phone: 212.659.5549 |
fax 212.659.8539|
patient line 212.659.5429
E Mail: william.oh@mssm.edu

Title: **Phase II randomized trial of enzalutamide/MDV-3100 and LHRH analogue vs combined androgen deprivation (LHRH analogue + bicalutamide) in metastatic hormone sensitive prostate cancer**

STUDY SCHEMA 6

1.0 OBJECTIVES 7

2.0 BACKGROUND 7

3.0 DRUG INFORMATION 12

4.0 ELIGIBILITY CRITERIA 19

5.0 TREATMENT PLAN 21

6.0 TOXICITIES TO BE MONITORED AND DOSAGE MODIFICATION 23

7.0 STUDY CALENDAR 25

8.0 CRITERIA FOR EVALUATION AND ENDPOINT DEFINITION 26

9.0 CRITERIA FOR REMOVAL FROM STUDY 30

10.0 CORRELATIVE STUDIES 30

11.0 STATISTICAL CONSIDERATIONS 35

12.0 ADVERSE EVENTS REPORTING 40

13.0 REFERENCES 47

14.0 DATA AND SAFETY MONITORING PLAN 51

15.0 APPENDICES 52

STUDY SCHEMA

Non castrate metastatic prostate cancer
$\leq$ 3 months of androgen deprivation for metastatic disease
At least 6 months since prior androgen deprivation therapy

↓

PS - 0 - 2,
Adequate marrow, hepatic & renal function
No history of seizures or risk factors for seizures

↓

STRATIFY by race (African American or Other) and by bone pain (yes or no)

(yields 4 strata)

R A N D O M I Z E within strata

Arm A	Arm B
**LHRH analogue**	**LHRH analogue**
**+**	**+**
**Enzalutamide**	**Bicalutamide**
**160 mg orally daily**	**50 mg orally daily**

↓

DETERMINE PRIMARY ENDPOINT:

% of patients achieving a PSA nadir of $\leq$ 4 ng/ml at month 7
(Patients should continue on study therapy after month 7 unless any of the criteria in section 9.0 for withdrawal are met)

1.0 OBJECTIVES:

Primary objective:

To compare the rates of achieving PSA remission at month 7 with LHRH analogue therapy and enzalutamide (Arm A) with that achieved with LHRH analogue and bicalutamide (Arm B) in metastatic hormone sensitive prostate cancer .

Secondary Objectives:

1) To compare the primary endpoint by race.

2) To compare the rates of each of 2 types of response by treatment arm:
   a) measurable disease response; and
   b) PSA response.

3) To compare each of 7 time-to-event endpoints by treatment arm:
   a) duration of overall response (RD);
   b) duration of stable disease (SDD);
   c) time to treatment failure (TTF);
   d) time-to-progression (TTP);
   e) TTP in patients with bone metastases;
   f) progression-free survival (PFS); and
   g) overall survival (OS).

4) To compare the rates of each type of toxicity by treatment arm.

5) To compare the incidence rate of skeletal related events (SRE), and the time until SRE, separately by treatment arm.

6) To compare the rates of CTC response by treatment arm..

7) To explore the molecular mechanisms within the androgen receptor pathway by determining the levels of CXCR4 and TMPRSS2-ERG expression, androgen metabolism enzymes, androgen receptor variants, and length of CAG repeats within the androgen receptor gene, and to associate them with the primary endpoint.

2.0 BACKGROUND:

Androgen deprivation therapy (ADT) remains the most effective frontline therapy for metastatic prostate cancer. Despite this, a proportion of patients demonstrate inadequate response to combined androgen blockade (CAB) and demonstrate worse clinical outcomes. The extent of response to ADT is reportedly a predictor of OS and PFS outcome related to prostate cancer. The Southwest Oncology Group (SWOG) randomized clinical trial evaluating continuous versus intermittent ADT in metastatic prostate cancer has completed accrual and Hussain et al [1] detail

the prognostic importance of PSA nadir ≤4ng/ml during the first 7 months of induction of ADT. Of the total patients enrolled on SWOG 9346 (INT-0162) study, about 52-57% were eligible for randomization after achieving a PSA level of ≤4 ng/ml with stable or declining levels at months 6 and 7 after CAB. The median survival was 13 months in the group with PSA nadir > 4 ng/ml, 44 months in the group with PSA nadir between 4 ng/ml to 0.2 ng/ml and 75 months in the patients with undetectable (<0.1 ng/ml). These differences present a great opportunity for use of this surrogate endpoint to predict overall disease related outcome. Now the final results of SWOG 9346 have been reported [2] and reveal that continuous androgen suppression likely confers an OS benefit over intermittent androgen deprivation and establishes this as the standard control arm for future trials in metastatic prostate cancer. The results of SWOG 9346 also reveal that despite best efforts about half of the patients do not achieve optimal outcome with initial androgen deprivation therapy. Hence there is an unmet need to improve on outcomes in metastatic prostate cancer earlier on, and subsequently delay the progression to the morbidity of castrate resistant prostate cancer. The androgen receptor inhibitor enzalutamide may present an opportunity to make early inroads in metastatic hormone sensitive prostate cancer.

Results of clinical investigations and studies on the molecular profiles of these progressing tumors show that the androgen receptor remains functional and that the tumors respond to strategies directed at the androgen receptor signaling axis. Overexpression of the androgen receptor has been documented in upwards of 50% of such prostate cancer specimens and is believed to contribute to tumor progression.[3, 4] In addition, currently approved anti-androgens have the potential to act as a partial agonist and stimulate androgen receptor signaling in the setting of androgen receptor overexpression, therefore exacerbating or accelerating tumor growth. The decline in serum levels of prostate-specific antigen (PSA) seen upon discontinuation of these agents is consistent with the agonist effects (“anti-androgen withdrawal syndrome”).

Enzalutamide (MDV3100/ Xtandi: Medivation/Astellas Inc) is an androgen receptor (AR) signaling inhibitor rationally designed to block multiple steps in the AR signaling pathway and to be devoid of agonist activity. Enzalutamide inhibits androgen-induced receptor activation (binding of androgens to ARs in the cytosol), inhibits nuclear translocation of activated ARs, and inhibits the association of the activated AR with chromatin, even in the setting of AR overexpression and in prostate cancer cells resistant to anti-androgens such as bicalutamide. The consequence of enzalutamide AR signaling inhibition is decreased growth of prostate cancer cells, induction of cancer cell death, and tumor regression.

The apoptotic effect of enzalutamide is consistent with AR blockade. It also induces cleavage of poly adenosine diphosphate-ribose polymerase (PARP), hence causing apoptosis, in prostate cancer cells, while bicalutamide treatment has no effect on PARP cleavage. In a mouse xenograft model of castration-resistant prostate cancer enzalutamide dose-dependently reduced tumor volume while bicalutamide treatment showed no significant benefit. Safety pharmacology studies in mice, rats, and dogs were performed with MDV3100 to assess any acute effects on central nervous system, respiratory, and cardiovascular parameters. No enzalutamide -related effects were noted in the central nervous system study assessing a functional observation battery and in a respiratory study in rats. Enzalutamide inhibits the human ether-a-go-go-related gene (hERG) channel; however, the highest free concentrations of MDV3100 expected in patient plasma at a steady-state dose of 160 mg/day are well below the hERG inhibitory concentration 50% (IC50)

value. No drug –related effects on cardiac electrophysiology were noted in a safety pharmacology study in conscious, telemetered dogs.

As enzalutamide inhibits the gamma amino butyric acid (GABA)-gated chloride channel, convulsion potential was assessed in single- and multiple-dose studies in mice. Oral therapy daily for 7 days was associated with convulsions in a dose-dependent manner with doses ≥ 200 mg/kg being active. When administered as a single dose of 400 mg/kg the treatment was also associated with convulsions. At the highest dose at which no convulsions occurred (single dose, 100 mg/kg), the maximum plasma concentration (Cmax) and area under the curve at 24 hours after dosing (AUC24) were at least 2.5-times higher than those in patients receiving 160 mg/day. The incidence of seizures was noted in 0.6% of the patients treated in the registration trial. 5 of 800 patients experienced seizures on enzalutamide therapy and none on placebo therapy. The seizures were reversible after therapy was discontinued [5]. One case of status epilepticus (confusion associated with partial complex-status epilepticus) required medical intervention; the four other seizures were self-limited and did not recur after study-drug discontinuation. Four of the seizures were witnessed. Potentially predisposing factors were present in several patients. Two patients had brain metastases, 1 of whom had a seizure reported 26 days after the last dose of enzalutamide. One patient had inadvertently been administered lidocaine intravenously immediately before the seizure, and 1 patient with brain atrophy had an unwitnessed event classified as a seizure, in the context of a history of heavy alcohol use, after initiation of haloperidol 7 days beforehand. One additional adverse event reported by the investigator as syncope had several features suggestive of seizure. The enzalutamide group had a lower incidence of adverse events of grade 3 or above (45.3%, vs. 53.1% in the placebo group). The median time to the first such adverse event was 12.6 months in the enzalutamide group, as compared with 4.2 months in the placebo group. There was a higher incidence of all grades of fatigue, diarrhea, hot flashes, musculoskeletal pain, and headache in the enzalutamide group than in the placebo group. Cardiac disorders were noted in 6% of patients receiving enzalutamide and in 8% of patients receiving placebo (with cardiac disorders of grade 3 in 1% and 2%, respectively). Hypertension or increased blood pressure was observed in 6.6% of patients in the enzalutamide group and 3.3% of those in the placebo group.

There were no significant between-group imbalances in the rates of other adverse events, such as hyperglycemia, weight gain, hyperlipidemia, or glucose intolerance. Therefore, there was no evidence to suggest the development of a metabolic syndrome associated with enzalutamide, although the study was not designed to formally evaluate this event. Liver-function abnormalities were reported as adverse events in 1% of patients receiving enzalutamide and in 2% of those receiving placebo.

Clinical data with enzalutamide

The study that led to the FDA approval of enzalutamide was a double blind placebo controlled phase III randomized trial. The study revealed an OS benefit with enzalutamide with median survival of 18.4 months as compared to median survival of 13.6 months with placebo ($p<0.0001$) [5]. The phase I/II study showed very promising efficacy in the chemotherapy naïve patient population with PSA response rates at 62% and time to progression of 41 weeks [6]. A placebo controlled randomized trial called PREVAIL in castrate resistant, chemotherapy naïve patient population has been conducted and completed accrual. The results are awaited but to date after

completing enrollment of 1680 patients no significantly increased toxicities have been noted.

Androgen deprivation therapy in metastatic prostate cancer

Testosterone suppression remains the mainstay of initial therapy in metastatic prostate cancer. The addition of bicalutamide confers a small but finite improvement in OS outcome in metastatic ca prostate. The resistance mechanisms to bicalutamide are attributed to the agonistic effect. Enzalutamide has no agonist effect on the androgen receptor and has PARP inhibition effects. The mechanism of action of enzalutamide, along with the proven clinical efficacy in metastatic castrate resistant disease, provides a rationale to utilize enzalutamide in an earlier setting of hormone sensitive prostate cancer.

The results of the CHAARTED (ECOG led) trial [13] were recently presented at the annual ASCO meeting in June 2014. The study randomized patients with metastatic prostate cancer to receive androgen deprivation therapy +/- docetaxel chemotherapy. The patients on the docetaxel arm received a maximum of 6 cycles of docetaxel at a dose of 75mg/m2 and were started on therapy within 4 months of initiating androgen deprivation therapy. Patients were stratified based on disease volume; low volume and high volume. The latter was defined as the presence of visceral metastases and/ or 4 or more bone metastases. 64% of the patients on the hormone therapy only arm and 67% on the docetaxel arm had high volume disease. The overall survival results reveal an OS benefit with the addition of docetaxel (Hazard ratio 0.63, p= 0.0006) with median survival of 42.3 and 52.7 months in the hormone and chemotherapy arms respectively. A median survival improvement of 17 months was noted in the high volume disease patients. (Median survival of 32.2 months and 49.2 months). Within the low volume disease patient population the OS results have not been reported. The above results have changed the standard of care to incorporate docetaxel based chemotherapy in the management of high volume hormone sensitive metastatic prostate cancer. Based on these results and remaining cognizant of the changing standard of care we will amend the eligibility criteria of the protocol to include patients with metastatic disease who either have

a) low volume disease

or

b) patients who are not candidates for docetaxel chemotherapy

or

c) Patients refusing docetaxel chemotherapy.

Race in prostate cancer

African American (AA) men are 1.6 times more likely to be diagnosed with prostate cancer and 2.4 times more likely to die of the disease as compared to Caucasian (CA) men. The disparity is not entirely related to access to care as was shown by a study conducted in the Veterans Health system which found an OS disparity between AA and CA patients[7]. It appears that African American patients had a lower chance of maintaining the PSA nadir of 4ng/ml or less as compared to the entire group and are likely to have shorter progression free survival.[8] Even in patients

treated with hormone therapy for advanced prostate cancer on clinical trials the overall survival outcome was noted to be shorter in patients of AA racial origin in both SWOG and RTOG trials. [9,10]

The exact causes of this racial disparity are unknown; a number of factors such as diet, comorbid conditions and genetic factors have been cited as possible causes. Specifically the length of CAG repeats on the androgen receptor gene has been reported to be significantly different in association with AA race as compared to CA origin [11**]**. In addition racial differences have also been found among variants of the genes of the enzymes involved in androgen biosynthesis and metabolism, such as SRD5A2, CYP17, and CYP3A4. The levels of expression and CAG repeat length of AR also show racial divergence and may be critical molecular alterations for racial disparity. Growth factors and their receptors, which promote cancer cell growth, are another potential cause of the disparity; both EGFR and EPHB2, two of the most studied receptors, show interethnic differences. Differences have also been found among genes regulating cell apoptosis, such as BCL2, which is increased in prostate cancer in the AA population.[12]

The androgen receptor inhibitor MDV-3100/enzalutamide (Medivation Inc and Astellas Inc) has revealed very promising activity in metastatic castrate resistant prostate cancer pretreated with docetaxel therapy. The agent revealed an OS benefit as compared to placebo in a double blind randomized study. In phase III trials conducted of enzalutamide, both pre and post chemotherapy, the number of patients with African American origin was very small and hence the effects of the differences in efficacy, if any, have not been studied. This proposal enables an assessment of a minority patient population that is reported to have a clinically worse outcome and explores the possibility of improving outcome and response by using a more effective androgen receptor inhibitor such as enzalutamide. Also this study will plan on tissue and serum collection to explore the molecular mechanisms within the androgen receptor pathway by determining the levels of CXCR4 and TMPRSS2-ERG expression, androgen metabolism enzymes, androgen receptor variants, and length of CAG repeats within the androgen receptor gene, and other correlative testing to explore the scientific reasons for the disparity.

Clinical correlates:

Bio-specimens: Baseline tumor tissue biopsies (bone is preferable but other sites permitted if patient has no accessible bone metastases for biopsy) will be mandatory for study enrollment. Post treatment core biopsies of bone tumor tissues will be encouraged and obtained from the patients in both arms.

Biomarkers: CXCR4, ERG and key androgen biosynthesis enzymes (SRD5A2, CYP17, CYP3A4, AKR1C3 and HSD17b6) will be analyzed in bio specimens.

Background and rationale: CXCR4 and TMPRSS2-ERG fusion genes were shown to be differentially regulated in human tumor tissues in African American and Caucasian men. CXCR4 has been shown to be associated with aggressive disease and could possibly be the reason for racial disparity in outcomes noted in African American men.TMPRSS2-ERG fusions were reported to be significantly localized to prostate tumors in Caucasian men. CXCR4 expression has been shown to be a key molecular predictor of bone metastasis. TMPRSS2-ERG fusion status is not known in bone metastasis. Published data demonstrate that TMPRSS2-ERG fusions are early markers for prostate cancer, but their expression is sustained in advanced cancers suggesting that fusion activated ERG factor is still functional in tumor cells. Both

CXCR4 and TMPRSS2-ERG are androgen responsive genes in prostate tumor cells. Androgen biosynthesis enzyme expression has been shown to be persistent in advanced patients, who undergo hormonal therapy. We will analyze the expression of key enzymes (SRD5A2, CYP17, CYP3A4, AKR1C3 and HSD17b6) contributing to intratumoral androgen biosynthesis and test if the levels of these enzymes has an impact on response to ADT in either of the therapeutic arms of the study. Pre and post tumor tissues in both arms of the study will be analyzed for marker expression.

3.0 DRUG INFORMATION:

3.1 MDV-3100/Enzalutamide/ (Xtandi) : Astellas/Medivation Inc. (Study Drug)

For patients enrolled and treated on this clinical trial, and randomized to Arm A, the above medication will be supplied by Medivation/Astellas Inc and dispensed in the treatment facility.

DESCRIPTION

Enzalutamide is an androgen receptor inhibitor. The chemical name is 4-{3-[4-cyano-3-(trifluoromethyl)phenyl]-5,5-dimethyl-4-oxo-2-sulfanylideneimidazolidin-1-yl}-2-fluoro-*N*-methylbenzamide.The molecular weight is 464.44 and molecular formula is C21H16F4N4O2S. Enzalutamide is a white crystalline non-hygroscopic solid. It is practically insoluble in water. XTANDI is provided as liquid-filled soft gelatin capsules for oral administration. Each capsule contains 40 mg of enzalutamide as a solution in caprylocaproyl polyoxylglycerides. The inactive ingredients are caprylocaproyl polyoxylglycerides, butylated hydroxyanisole, butylated hydroxytoluene, gelatin, sorbitol sorbitan solution, glycerin, purified water, titanium dioxide, and black iron oxide.

CLINICAL PHARMACOLOGY

Mechanism of Action

Enzalutamide is an androgen receptor inhibitor that acts on different steps in the androgen receptor signaling pathway.

Enzalutamide has been shown to competitively inhibit androgen binding to androgen receptors and inhibit androgen receptor nuclear translocation and interaction with DNA. A major metabolite, N-desmethyl enzalutamide, exhibited similar *in vitro* activity to enzalutamide. Enzalutamide decreased proliferation and induced cell death of prostate cancer cells *in vitro*, and decreased tumor volume in a mouse prostate cancer xenograft model.

Pharmacokinetics

The pharmacokinetics of enzalutamide and its major active metabolite (N-desmethyl enzalutamide) were evaluated in patients with metastatic castration-resistant prostate cancer and healthy male volunteers. The plasma enzalutamide pharmacokinetics are adequately described by a linear two-compartment model with first-order absorption.

Absorption

Following oral administration (XTANDI 160 mg daily) in patients with metastatic castration-resistant prostate cancer, the median time to reach maximum plasma enzalutamide concentrations (Cmax) is 1 hour (range 0.5 to 3 hours). At steady state, the plasma mean Cmax values for enzalutamide and N-desmethyl enzalutamide are 16.6 μg/mL (23% CV) and 12.7 μg/mL (30%

CV), respectively, and the plasma mean predose trough values are 11.4 µg/mL (26% CV) and 13.0 µg/mL (30% CV), respectively.

With the daily dosing regimen, enzalutamide steady state is achieved by Day 28, and enzalutamide accumulates approximately 8.3-fold relative to a single dose. Daily fluctuations in enzalutamide plasma concentrations are low (mean peak-to-trough ratio of 1.25). At steady state, enzalutamide showed approximately dose proportional pharmacokinetics over the daily dose range of 30 to 360 mg.

A single 160 mg oral dose of XTANDI was administered to healthy volunteers with a high-fat meal or in the fasted condition. A high-fat meal did not alter the AUC to enzalutamide or N-desmethyl enzalutamide.

Distribution and Protein Binding

The mean apparent volume of distribution (V/F) of enzalutamide in patients after a single oral dose is 110 L (29% CV). Enzalutamide is 97% to 98% bound to plasma proteins, primarily albumin. N-desmethyl enzalutamide is 95% bound to plasma proteins.

Metabolism

Following single oral administration of 14C-enzalutamide 160 mg, plasma samples were analyzed for enzalutamide and its metabolites up to 77 days post dose. Enzalutamide, N-desmethyl enzalutamide, and a major inactive carboxylic acid metabolite accounted for 88% of the 14C-radioactivity in plasma, representing 30%, 49%, and 10%, respectively, of the total 14C-AUC0-inf.

In vitro, human CYP2C8 and CYP3A4 are responsible for the metabolism of enzalutamide. Based on *in vivo* and *in vitro* data, CYP2C8 is primarily responsible for the formation of the active metabolite (N-desmethyl enzalutamide).

Elimination

Enzalutamide is primarily eliminated by hepatic metabolism. Following single oral administration of 14C-enzalutamide 160 mg, 85% of the radioactivity is recovered by 77 days post dose: 71% is recovered in urine (including only trace amounts of enzalutamide and N-desmethyl enzalutamide), and 14% is recovered in feces (0.4% of dose as unchanged enzalutamide and 1% as N-desmethyl enzalutamide).

The mean apparent clearance (CL/F) of enzalutamide in patients after a single oral dose is 0.56 L/h (range 0.33 to 1.02 L/h).

The mean terminal half-life (t1/2) for enzalutamide in patients after a single oral dose is 5.8 days (range 2.8 to 10.2 days).

Following a single 160 mg oral dose of enzalutamide in healthy volunteers, the mean terminal t1/2 for N-desmethyl enzalutamide is approximately 7.8 to 8.6 days.

Drug Interactions

Effect of Other Drugs on XTANDI:

In a drug-drug interaction trial in healthy volunteers, a single 160 mg oral dose of XTANDI was administered alone or after multiple oral doses of gemfibrozil (strong CYP2C8 inhibitor). Gemfibrozil increased the AUC0-inf of enzalutamide plus N-desmethyl enzalutamide by 2.2-fold with minimal effect on Cmax.

In a drug-drug interaction trial in healthy volunteers, a single 160 mg oral dose of XTANDI was

administered alone or after multiple oral doses of itraconazole (strong CYP3A4 inhibitor). Itraconazole increased the AUC0-inf of enzalutamide plus N-desmethyl enzalutamide by 1.3-fold with no effect on Cmax. The effects of CYP2C8 and CYP3A4 inducers on the exposure of XTANDI have not been evaluated *in vivo*.

CLINICAL STUDIES

The efficacy and safety of XTANDI in patients with metastatic castration-resistant prostate cancer who had received prior docetaxel-based therapy were assessed in a randomized, placebo-controlled, multicenter phase 3 clinical trial. The primary endpoint was overall survival. A total of 1199 patients were randomized 2:1 to receive either XTANDI orally at a dose of 160 mg once daily (N = 800) or placebo orally once daily (N = 399). All patients continued androgen deprivation therapy. Patients were allowed, but not required to continue or initiate glucocorticoids. Study treatment continued until disease progression (evidence of radiographic progression, a skeletal-related event, or clinical progression), initiation of new systemic antineoplastic treatment, unacceptable toxicity, or withdrawal. Patients with a history of seizure, taking medicines known to decrease the seizure threshold, or with other risk factors for seizure were not eligible

The following patient demographics and baseline disease characteristics were balanced between the treatment arms. The median age was 69 years (range 41-92) and the racial distribution was 92.7% Caucasian, 3.9% Black, 1.1% Asian, and 2.1% Other. Ninety-two percent of patients had an ECOG performance status score of 0-1 and 28% had a mean Brief Pain Inventory score of ≥ 4. Ninety-one percent of patients had metastases in bone and 23% had visceral involvement in the lung and/or liver. Fifty-nine percent of patients had radiographic evidence of disease progression and 41% had PSA-only progression on study entry. All patients had received prior docetaxel-based therapy and 24% had received two cytotoxic chemotherapy regimens. During the trial, 48% of patients on the XTANDI arm and 46% of patients on the placebo arm received glucocorticoids.
The pre-specified interim analysis at the time of 520 events showed a statistically significant improvement in overall survival in patients on the XTANDI arm compared to patients on the placebo arm.

HOW SUPPLIED/STORAGE AND HANDLING

□ XTANDI (enzalutamide) 40 mg capsules are supplied as white to off-white oblong soft gelatin capsules imprinted in black ink with MDV. XTANDI capsules are available in the following package sizes:
□ Bottles of 120 capsules (NDC 0469-0125-99)
Recommended storage: Store XTANDI capsules at 20°C to 25°C (68°F to 77°F) in a dry place and keep the container tightly closed.
Excursions permitted from 15°C to 30°C (59°F to 86°F).

PATIENT COUNSELING INFORMATION

See FDA-approved patient labeling (PATIENT INFORMATION).
□ Instruct patients to take their dose at the same time each day (once daily). XTANDI can be taken with or without food. Each capsule should be swallowed whole. Do not chew, dissolve, or open the capsules.
□ Inform patients that XTANDI has been associated with an increased risk of seizure. Discuss conditions that may predispose to seizures and medications that may lower the seizure threshold.

Advise patients of the risk of engaging in any activity where sudden loss of consciousness could cause serious harm to themselves or others.

□ Inform patients that XTANDI may cause dizziness, mental impairment, paresthesia, hypoesthesia, and falls.

□ Inform patients that they should not interrupt, modify the dose, or stop XTANDI without first consulting their physician.

Inform patients that if they miss a dose, then they should take it as soon as they remember. If they forget to take the dose for the whole day, then they should take their normal dose the next day. They should not take more than their prescribed dose per day.

□ Apprise patients of the common side effects associated with XTANDI: asthenia/fatigue, back pain, diarrhea, arthralgia, hot flush, peripheral edema, musculoskeletal pain, headache, upper respiratory infection, muscular weakness, dizziness, insomnia, lower respiratory infection, spinal cord compression and cauda equina syndrome, hematuria, paresthesia, anxiety, and hypertension.

PATIENT INFORMATION

□ Inform patients that XTANDI may be harmful to a developing fetus. Patients should also be informed that they should use a condom if having sex with a pregnant woman A condom and another effective method of birth control should be used if the patient is having sex with a woman of child-bearing potential. These measures are required during and for three months after treatment with XTANDI.

3.2 Bicalutamide (Casodex®) (NSC-722665)

For bicalutamide, goserelin acetate, and leuprolide acetate, commercially Available preparations are used as per the FDA approved dose, route and schedule. Information about commercial drugs is publicly available in the Physician's Desk Reference (PDR), prescribing information and other resources.

Chemistry: Bicalutamide is a racemic mixture containing two enantiomers, (2RS)-4'-Cyano-3(4-fluorophenylsulphonyl)-2-hydroxy-2-methyl-3'-trifluoromethyl) propionanilide. Bicalutamide is an active non-steriodal antiandrogen and its antiandrogen activityresides exclusively in the (-) or (R) enantiomer. Unlike flutamide, it is peripherally selective and does not cause a rise in serum LH or Testosterone in male rats and dogs. This peripheral selectivity may be because it penetrates poorly the CNS and Hypothalamus (the site of negative feedback of androgens). In humans, rises in LH, Testosterone and Estradiol concentrations were seen. These rises were not dose related. In 90%, testosterone levels remained within normal limits. There was no significant rise in mean serum FSH. Bicalutamide has been given to over 3,500 men in 35 different clinical studies worldwide, in doses up to 600 mg daily.

Adverse Effects:

When bicalutamide is given in combination with an LHRH analog, the pharmacologic adverse event profile is dominated by the LHRH analog and includes hot flashes (53%), gynecomastia (9%) and breast pain (6%). Other adverse events reported regardless of causality included diarrhea (12%), constipation (22%), nausea (15%) and abdominal pain (11%). Other adverse

were reported, such as fatigue (22%), pain (35%), back pain (25%), pelvic pain (21%), infection (18%), peripheral edema (13%), dyspnea (13%), nocturia (12%), hematuria (12%), anemia (11%), dizziness (10%). Bicalutamide has been associated with changes in liver function, although these are infrequent (7%) and rarely occur with jaundice. Many of these changes improved or resolved despite continuation of bicalutamide therapy. There have been no reports of fatal hepatotoxicity associated with bicalutamide therapy.

Pharmacology

Pharmacokinetics:

Animal studies: After oral single dose administration, absorption of the compound was slow with peak concentration occurring 3 - 12 hours and plateau between 2 and 48 hours. There was non-proportional increase in plasma levels with increasing doses. Elimination half life ranges from 17 - 28 hours in male rats, 21 - 29 hours in female rats and 5 - 7.5 days in dogs. 91 - 96% of bicalutamide is bound to plasma protein.

Human studies: After single doses, mean time for peak plasma concentration was 6 hours at 10 and 30 mg, but at 50 mg, it was 16 hours. Mean plasma elimination half-lives after 12 weeks of 10, 30, 50, 100 mg/day was 7 - 10 days. This finding was consistent with single dose data. In patients given daily doses of 50 mg, mean plasma concentration was 10 ug/ml at 12 weeks. After single doses, there was linear increase with doses between 10 and 50 mg, but became non-linear at doses of 50 - 100 mg. At 100 mg, the oral bioavailability is reduced by 30% but plasma elimination half-life is unchanged. Bicalutamide is extensively metabolized and metabolites are excreted by both the biliary and urinary system.

Formulation: Bicalutamide is prepared as round, film-coated green or white tablets containing standard recipients and 50 mg of the drug.

Storage and stability: All packages of bicalutamide should be stored securely in a dry place at room temperature.

Route of Administration: Bicalutamide is to be administered in tablet form as a 50 mg once-daily oral dose. Patients should be instructed to take one tablet once daily.

3.3 Gonadotropin/Luteinizing Hormone Releasing Hormone (GNRH/LHRH) analogues:

The commonly used preparations are listed below. However any of the other preparations with FDA approval that result in androgen suppression are acceptable.

Goserelin acetate implant (Zoladex®) / Leuprolide (Lupron)

a. PHARMACOLOGY

Mechanism of Action: Following initial administration in males, goserelin causes an initial increase in serum luteinizing hormone (LH) and follicle stimulating hormone (FSH) levels with subsequent increases in serum levels of testosterone.

Chronic administration of goserelin leads to sustained suppression of pituitary gonadotropins, and serum levels of testosterone consequently fall into the range normally seen in surgically castrated men approximately 2-4 weeks after initiation of therapy.

b. PHARMACOKINETICS

1. Absorption: Goserelin 3.6 mg is released slowly in first 8 days, and then rapid and

continuous release for the remainder of the 28 day dosing period. Time to peak concentration for goserelin 3.6 mg is 12-15 days in males and 8-22 days in females. Goserelin 10.8 mg exhibits an initial rapid release resulting in a peak concentration at 2 hours after dosing. From Day 4 until the end of the 12-week dosing interval, the sustained release of goserelin produces a reasonably stable systemic exposure.

2. Distribution: Apparent volumes of distribution determined after subcutaneous administration of 250 mcg aqueous solution of goserelin were 44.1 and 20.3 liters for males and females, respectively. Goserelin is approximately 27% protein bound.

3. Metabolism: Metabolism of goserelin by hydrolysis of the C-terminal amino acids is the major clearance mechanism. The half-life elimination (t1/2)) is approximately 4 hours in males and 2 hours in females

4. Elimination: Clearance of goserelin is very rapid and occurs primarily via urinary excretion (>90%; 20% as unchanged drug).

c. ADVERSE EFFECTS

1. Refer to the package insert or manufacturer website for the most complete and up to date information on contraindications, warnings and precautions, and adverse reactions.

Side Effects

Adverse events associated with the use of Leuprolide/Goserelin may include, but are not limited to, the following:

- hot flashes
- injection site reactions
- weight gain
- increase in liver enzymes
- tiredness
- hypertension
- back and joint pain
- chills
- urinary tract infection
- decreased sex drive and trouble with erectile function

Adverse effects occurring in <1%, postmarketing, and/or case reports:

1. ALT increased, anaphylaxis, AST increased, diabetes, glucose tolerance decreased, hypercalcemia, hypercholesterolemia, hyperlipidemia, hypersensitivity reactions, hypotension, ovarian cyst, pituitary apoplexy, psychotic disorders, urticaria.
2. Pregnancy and Lactation: Not applicable as this study only enrolls male patients. Pregnancy category X in patients with endometriosis and endometrial thinning. Pregnancy category D in patients with advanced breast cancer. It is not known if goserelin is excreted in human

milk, however goserelin is excreted in the milk of lactating rats.

3. Drug Interactions: Luteinizing hormone-releasing hormone analogs may diminish the therapeutic effect of antidiabetic agents. No formal drug-drug interaction studies have been performed. Please refer to the current FDA-approved package insert for additional information.
4. The FDA issued a safety communication in October 2010 based on their ongoing safety review of LHRH agonists. The safety communication discusses the potential for an increased risk of diabetes and cardiovascular disease (myocardial infarction, sudden cardiac death, stroke) associated with these agents. The risk is thought to be low in men receiving LHRH agonists for prostate cancer. In this trial, LHRH agonists are being administered for a short period of time. FDA recommendations include management of cardiovascular risk factors according to current standards of practice.

d. DOSING & ADMINISTRATION

1. Dosing – See Package Insert of preparation used.
2. Goserelin is administered subcutaneously into the anterior abdominal wall below the navel line using aseptic technique.

e. STORAGE & STABILITY

Refer to the current FDA-approved package insert for storage, stability and special handling information.

f. HOW SUPPLIED

1. Goserelin acetate implant is available in a 3.6 mg or 10.8 mg disposable syringe device. The unit is sterile and comes in a sealed, light- and moisture-proof, aluminum foil laminate pouch containing a desiccant capsule.
2. Goserelin is commercially available and will not be supplied. Refer to the current FDA-approved package insert for additional information.

Leuprolide (Eligard®, Lupron Depot®) (NSC-377526)

PHARMACOLOGY

Mechanism of Action: Leuprolide inhibits gonadotropin secretion by acting as a luteinizing hormone-releasing hormone (LHRH) agonist. Continuous administration results in suppression of ovarian and testicular steroidogenesis due to decreased levels of LH and FSH with subsequent decrease in testosterone (male) and estrogen (female) levels. In males, testosterone levels are reduced to below castrate levels. Leuprolide may also act directly on the testes as well as act by a different mechanism not directly related to reduction in serum testosterone.

DOSING & ADMINISTRATION

1. Dosing – Per package insert. Typically 7.5 mg monthly or 22.5 mg every 3 months
2. Leuprolide is administered intramuscular (Lupron Depot®) or subcutaneous (Eligard®) injection based on commercial depot formulation. Injection sites should be varied periodically.

STORAGE & STABILITY

Refer to the current FDA-approved package insert for storage, stability and special handling information.

HOW SUPPLIED

1. Leuprolide acetate is available in 3.75 mg, 7.5 mg, 11.25 mg, 22.5 mg, 30mg, or 45 mg depot formulation kit with accompanying diluent. The prefilled dual chamber syringe contains lyophilized microspheres of leuprolide acetate incorporated in a biodegradable lactic acid polymer.
2. Leuprolide/Goserelin is commercially available and will not be supplied.

Other LHRH agents can be utilized per the FDA approved dose and schedule. GNRH antagonists cannot be used.

4.0 ELIGIBILITY CRITERIA

4.1 Inclusion Criteria:

1. Histologically confirmed prostate adenocarcinoma with metastasis either starting or recently started on LHRH analogue therapy. [Late induction permitted within 3 months of starting LHRH analogue therapy or antiandrogen] All patients who have not initiated hormone therapy (Early induction patients) must have elevated PSA ≥ 4 ng/ml within 28 days prior to registration. For late induction registrations, PSA must be ≥ 4 ng/ml prior to start of androgen deprivation therapy; either antiandrogen or LHRH analogue or GNRH antagonist .If patients are on antiandrogen, this will need to be discontinued for at least 7 days prior to registration.
2. Patients with a history of prior neoadjuvant or adjuvant hormone therapy are eligible provided they have received twenty four or less months of hormone treatment (single or combination treatment, excluding orchiectomy). Neoadjuvant/adjuvant hormone therapy must have been discontinued at least 6 months prior to registration. This is intended to exclude patients who might have been rendered indirectly androgen insensitive.
3. There must be no plans to receive concomitant chemotherapy, biological response modifiers, radiation therapy or hormonal therapy. Concomitant radiation therapy is allowed for the palliation of severe pain/neuropathic compression.
4. Prior or concomitant use of megestrol acetate for the treatment of hot flashes is allowed.
5. Patients must have a performance status of 0 - 2 by Zubrod Criteria.
6. Patients must have recovered from any major infections and/or surgical procedures and, in the opinion of the investigator, not have significant active medical illness precluding protocol treatment or survival.
7. No prior malignancy is allowed except for adequately treated basal cell (or squamous cell) skin cancer, superficial or in situ cancer of the bladder. For an invasive cancer the patients should be disease free for at least 3 years prior to enrollment on study.
8. For all patients a bone scan must be performed within 60 days prior to registration for tumor assessment. CT scans (abdomen and pelvis) and chest x-ray are optional, but must be repeated if used for disease assessment. For late induction registrations, tumor assessment imaging showing metastatic disease must be available prior to start of androgen deprivation therapy.

9. Age 18 or older and willing and able to provide informed consent.
10. Willingness to swallow pills and no medical condition that would interfere with this.
11. Male patient and his female partner who is of childbearing potential must use 2 acceptable methods of birth control (one of which must include a condom as a barrier method of contraception) starting at screening and continuing throughout the study period and for 3 months after final study drug administration. Patients are also required to use a condom if having sex with a pregnant woman.
12. Patient should agree to a tumor tissue biopsy prior to protocol enrollment. Post therapy biopsy is optional.
13. Patients who are being treated with a GNRH antagonist should be willing to switch to a LHRH analogue after registration.
14. Patients must have one of the following a) Low volume disease (defined as no visceral metastases and < 4 bone metastases) or b) are not candidates for docetaxel based chemotherapy or 3) refused docetaxel chemotherapy

4.2 Patients must NOT meet any of the following exclusion criteria:

1. History of seizure or any condition that may predispose to seizure (e.g., prior cortical stroke, significant brain trauma) at any time in the past. Also, history of loss of consciousness or transient ischemic attack within 12 months of Day 1 visit;
2. Known or suspected brain metastasis or active leptomeningeal disease;
3. Severe concurrent disease, infection, or co-morbidity that, in the judgment of the Investigator, would make the patient inappropriate for enrollment;

4. Absolute neutrophil count < 1,000/μL, or platelet count < 50,000/μL, or hemoglobin<8 /dL) at the Screening visit.

5. Total bilirubin, alanine aminotransferase (ALT) or aspartate aminotransferase (AST) > 2.5 times the upper limit of normal

6. Creatinine > 177 μmol/L (2 mg/dL)

7. Clinically significant cardiovascular disease including:

□ Myocardial infarction within 6 months;

□ Uncontrolled angina within 3 months;

□ Congestive heart failure New York Heart Association (NYHA) class 3 or 4, or patients with history of congestive heart failure NYHA class 3 or 4 in the past, unless screening echocardiogram or multi-gated acquisition scan performed within 3 months results in a left ventricular ejection fraction that is ≥ 45%;

---History of clinically significant ventricular arrhythmias (e.g., ventricular tachycardia, ventricular fibrillation, torsades de pointes);

---History of Mobitz II second degree or third degree heart block without a permanent pacemaker in place;

---Hypotension as indicated by systolic blood pressure < 86 millimeters of mercury (mmHg) at the Screening visit;

---Bradycardia as indicated by a heart rate of < 50 beats per minute on the Screening ECG;

---Uncontrolled hypertension as indicated by systolic blood pressure > 170 mmHg or

diastolic blood pressure > 105 mmHg

8. Gastrointestinal disorder affecting absorption (e.g., gastrectomy, active peptic ulcer disease within last 3 months);
9. Treatment with concurrent 5-α reductase inhibitors (finasteride, dutasteride), estrogens, and/or cyproterone
10. Treatment with systemic biologic therapy for prostate cancer (other than approved bone targeted agents and GnRH-analogue therapy) or other agents with anti-tumor activity within 4 weeks of enrollment (Day 1 visit);
11. History of prostate cancer progression on ketoconazole;
12. Prior use, or participation in a clinical trial, of an investigational agent that blocks androgen synthesis (e.g., abiraterone acetate, TAK-700, TAK-683, TAK-448) or agents that block the androgen receptor (e.g., ARN-509)
13. Previous enzalutamide therapy;
14. Use of an investigational agent within 2 weeks of enrollment (Day 1 visit);
15. Use of herbal products that may have hormonal anti-prostate cancer activity and/or are known to decrease PSA levels (e.g., saw palmetto) or systemic corticosteroids greater than the equivalent of replacement steroids or > equivalent of 10 mg of prednisone per day within 4 weeks of enrollment (Day 1 visit);
16. Any condition or reason that, in the opinion of the Investigator, interferes with the ability of the patient to participate in the trial, which places the patient at undue risk, or complicates the interpretation of safety data.
17. Prior chemotherapy for metastatic disease.
18. ≥30 days of antiandrogen therapy monotherapy without androgen deprivation therapy.
19. Life expectancy of 6 months or less.

5.0 TREATMENT PLAN:

5.1 Patient Registration and Data Collection:

All patients shall be registered with the Cancer Center Clinical Trials Office at 313-576-9837 (Kimberlee Dobson).

At the time of registration, a pre-study form and all information required to verify eligibility shall be necessary on each patient prior to treatment. This form should be faxed to 313-576-8974 and eligibility verification will be sent within 48 hours. Data will be collected and maintained on study specific electronic case report forms in the Oncore Research Enterprise system at Karmanos Cancer Institute. Training and support will be provided for all staff entering data. Data entry must occur within 2 weeks of visits along with submission of applicable source documentation. The only concomitant medication data to be submitted is administration of bisphosphonate or denosumab.

5.2 Stratification and randomization procedures:

The registering data manager will first stratify the patient by race (AA or other) and cancer related bone pain at diagnosis of metastatic disease (present or absent) into one of 4 strata. Even if pain is well controlled/resolved at present the patient will be considered positive for bone pain. The patient will then be randomized to Arm A or Arm B using the stratum-appropriate randomization list prepared in advance by the study statistician Dr Lance Heilbrun. Arms A and

B are defined in the Study Schema (on page 5) and in Section 5.3 below. Each of the 4 randomization sequences will use random permuted blocks of varying sizes. The treatment arm randomization and enrollment confirmation will be communicated to site staff in writing within 24-48 hours from receipt of all eligibility source documents. Study treatment should start within 7 days of registration.

5.3 Study design and plan:

Overall Study Design and Plan
This study is a multicenter Phase II, randomized, screening study of oral MDV3100 (160 mg/day) in patients with metastatic hormone sensitive prostate cancer. Patients must not have received prior cytotoxic chemotherapy for metastatic disease. Radiation therapy, and initiation of bisphosphonates or other approved bone targeting agents, and standard of care steroid and pain management are allowed and should not result in discontinuation of study drug therapy.

For patients eligible for late induction registration the treatment start date will be the date of initiation of GNRH analogue or antagonist, with or without antiandrogen. Late induction patient visits will be adjusted based on the length of their prior hormone therapy. Study drug therapy should be continued as long as the patient is tolerating the study drug and continues androgen deprivation therapy or until confirmed radiographic disease progression or a skeletal related event AND one of the two following events: 1) initiation of another agent for treatment of prostate cancer. Or 2) Intolerance to therapy.

Patients will be randomized to receive either:

ARM A
Orchiectomy/LHRH analogue Leuprolide or Goserelin or any other FDA approved preparation, approved by PI on notification, except GNRH antagonist such as Degarelix) If patients are late induction and have started on degarelix (Firmagon;Ferring pharmaceuticals) or any GNRH antagonist they will need to be switched to a GNRH analogue.

and

Enzalutamide 160 mg (4Caps of 40 mg each) orally daily (experimental arm)

OR

ARM B
Orchiectomy/LHRH analogue Leuprolide or Goserelin or any other FDA approved preparation, approved by PI on notification, except GNRH antagonist such as Degarelix)
If patients are late induction and have started on degarelix(Firmagon;Ferring pharmaceuticals) or any other GNRH antagonist they will need to be switched to a LHRH/GNRH analogue.

and

Bicalutamide 50 mg orally daily (standard arm).

Stratification by ethnic origin (African American or other) and bone pain (Yes/No).

Patients should continue on study therapy after month 7 unless any of the criteria in section 9.0 for withdrawal are met

5.4 Agent Administration

Starting doses of the agents are as follows:

Enzalutamide is provided as 40 mg soft gelatin capsules in bottles with induction-sealed child-resistant caps.

Directions for Administration

Study drug doses should be taken as close as possible to the same time each day. All study patients on enzalutamide will take 4 capsules of study drug once daily unless dose adjustment has been done . Patients randomized to bicalutamide will take 1 tab of 50 mg daily dose. If dosing is missed on one day for any reason, double-dosing should NOT occur the following day. The oral medications can be taken with or without food. Documentation of daily dose will be conducted by patient on a study diary.

Storage and Labeling

Study drug should be stored in a secure location with limited access within the following temperature range: 59°F to 86°F (15°C to 30°C). Bottles will be labeled with the study protocol number, medication or bottle number, contents, directions for use, storage directions. Patients will be instructed to store study drug at room temperature out of the reach of children.

6.0 TOXICITIES TO BE MONITORED AND DOSAGE MODIFICATION:

All toxicities will be graded as per NCI Common Toxicity (CTCAE) version 4.0.

Toxicity evaluation will be done monthly for the first 8 visits starting with day 1 and after that every 3 months until progression. Radiologic evaluation at months 6 and 12 with scans (CT scans and bone scan), and PSA levels every month for the first 8 visits and after that every 3 months. After 12 months radiologic evaluation is required annually while patient is on study.

Dose Reduction/Dose Adjustment

Patients who experience a Grade 3 or greater toxicity that is attributed to study medication, and cannot be ameliorated by the use of adequate medical intervention should have their treatment interrupted until the toxicity improves to a Grade 2 or lower severity. Patients may subsequently be re-started on study drug, including at a reduced dose (80 mg daily of enzalutamide), with the written approval of the PI (or designee). **Any reports of seizure activity for patients randomized to enzalutamide require immediate and permanent discontinuation of this therapy.**

There are no dosage adjustments for hematologic toxicity.

Dosage adjustments for Non-hematologic Toxicity:

a. Diarrhea Toxicity:

The major toxic effect of bicalutamide is moderate diarrhea which is very rarely severe (exclusion of other causes of diarrhea should be considered in severe cases). The dose modifications for this will be as follows, if diarrhea is attributed to protocol therapy.
1. Grade 2 diarrhea, treat symptomatically with anti-diarrhea drugs.
2. Grade 2 diarrhea unresponsive to symptomatic treatment or Grade 3/4 diarrhea - discontinue bicalutamide/enzalutamide until complete recovery.

ABNORMAL LIVER FUNCTION TESTS (AST, ALT, BILIRUBIN)

Grade 2 or greater toxicity - Stop bicalutamide/enzalutamide; wait until liver function tests (LFTs) including AST/ALT and bilirubin are normal (Grade 1 or less). Restart treatment when AST/ALT and bilirubin return to normal/baseline. If toxicity (Grade 2 or worse) occurs again, discontinue protocol treatment.

Patients requiring treatment delay greater than 2 months for any reason should be removed from protocol treatment. Patients may complain of flatulence, bloating and mild "gas pains" which should not result in changes in treatment. Symptomatic treatment should be employed with antacids, simethicone, etc. Unexpected or fatal toxicities (including suspected reactions) assessed by the investigator to be related to study therapy/ies must be reported per serious adverse event reporting guidelines as mentioned.

7.0 STUDY CALENDAR: Visit dates and scans can be within +/- 7 days of planned dates. Study treatment must start within 7 days of registration. For patients eligible for late induction registration the treatment start date will be the date of initiation of GNRH analogue or antagonist, with or without antiandrogen.

Test	Pre study[1]	Day 1	Mth 1	Mth 2	Mth 3	Mth 4	Mth 5	Mth 6	Mth 7	Post Mth 7 Every 3mths	F/U[4] Every3 mths off study
Hand P	X	X	X	X	x			X	X	X	
PS	X	X	X	X	X			X	X	X	
Tox			X	X	X			X	X	X	
VS	X	X	X	X	X			X	X	X	
EKG	X										
CBC	X	X			X			X		X	
AST	X	X			X			X		X	
ALT	X	X			X			X		X	
Alk phos	X	X			X			X		X	
BUN	X	X			X			X		X	
lytes	X	X			X			X		X	
creat	X	X			X			X		X	
PT/INR[2]	X	X	X	X	X	X	X	X	X	X	
PSA	X	X	X	X	X	X	X	X	X	X	
Testosterone	X				X			X		X	
CXR/CT[3]	X							X			
Bone[3] scan	X							X			
OS											X
Progression		X	X	X	X	X	X	X	X		X
Tumor biopsy[5]	X[5]		X[5]								
CTC[5]		X[5]	X[5]								

1. Pre-study H & P, labs, EKG may be done within 28 days of registration. If Labs, and H and P are done within 72 hours prior to registration=they do not have to be repeated on day 1 of therapy unless clinically indicated.
2. PT, INR to be done only if patient on therapeutic Coumadin and then will need to be repeated at least once every month.
3. Tumor assessment scans must be within 60 days prior to registration for all patients. Bone scans are required for pretreatment and subsequent tumor assessments. CT/MRI of abdomen and pelvis should be done if necessary for tumor assessment. Radiologic assessment with bone scans should be done at 6 and 12 months on study (+/- 1 month) and then annually (+/- 1 month) as long as patient continues on study treatment. For late induction registrations, tumor assessment imaging showing metastatic disease must

be available prior to start of androgen deprivation therapy.

4. After completion of therapy patients will be followed every 3 months for disease status and survival.
5. Fresh tissue should be collected pre-therapy. Archival tissue should be submitted within 28 days of registration. Post treatment tumor biopsy (optional) and CTC can be conducted within 28-42 days after registration even for late induction patients. Late induction patients will get CTC at time of study entry and then 28-42 days later.
6. Disease progression is defined as evidence of radiographic progression, a skeletal-related event, or clinical progression. Progression of measurable disease is defined by RECIST 1.1 criteria, or progression of bone metastases or occurrence of new skeletal related event or by PSA per PCWG 2 criteria [Appendix B].

8.0 CRITERIA FOR EVALUATION AND ENDPOINT DEFINITION:

The RECIST 1.1 criteria with unidimensional measurement are to be used for measurable disease response evaluation [14].

Baseline Disease Assessment

Measurable lesions must be accurately measured in at least one dimension (longest diameter in the plane of measurement is to be recorded) with a minimum size of:

• 10 mm by CT scan (CT scan slice thickness no greater than 5 mm; when CT scans have slice thickness >5 mm, the minimum size should be twice the slice thickness).

• 10 mm caliper measurement by clinical exam (lesions which cannot be accurately measured with calipers should be recorded as nonmeasurable).

• 20 mm by chest X-ray.

• Malignant lymph nodes: To be considered pathologically enlarged and measurable, a lymph node must be ≥15 mm in short axis when assessed by CT scan (CT scan slice thickness is recommended to be no greater than 5 mm). At baseline and in follow-up, only the short axis will be measured and followed.

• Lytic bone lesions or mixed lytic-blastic lesions with identifiable soft tissue components that can be evaluated by cross-sectional imaging techniques such as CT or MRI can be considered measurable if the soft tissue component meets the definition of measurability described above.

• ‘Cystic lesions’ thought to represent cystic metastases can be considered measurable if they meet the definition of measurability described above.

However, if non-cystic lesions are present in the same patient, these are preferred for selection as target lesions.

Non-measurable lesions

Non-measurable lesions are all other lesions, including small lesions (longest diameter <10 mm or Pathological lymph nodes with 10 to <15 mm short axis), as well as truly non-measurable lesions.

• Blastic bone lesions are non-measurable.

• Lesions with prior local treatment, such as those situated in a previously irradiated area or in an area subjected to other loco-regional therapy, are usually not considered measurable unless there has been demonstrated progression in the lesion.

Target Lesions

• All measurable lesions up to a maximum of two lesions per organ and five lesions in total, representative of all involved organs, should be identified as target lesions and recorded and measured at baseline.

• Target lesions should be selected on the basis of their size (lesions with the longest diameter) and be representative of all involved organs, as well as their suitability for reproducible repeated measurements.

• All measurements should be recorded in metric notation using calipers if clinically assessed. A sum of the diameters (longest for non-nodal lesions, short axis for nodal lesions) for all target lesions

Non-target Lesions

All lesions (or sites of disease) not identified as target lesions, including pathological lymph nodes and all non-measurable lesions, should be identified as non-target lesions and be recorded at baseline. Measurements of these lesions are not required and they should be followed as ‘present’, ‘absent’ or in rare cases, ‘unequivocal progression’.

Evaluation of target lesions

Complete Response (CR): Disappearance of all target lesions. Any pathological lymph nodes (whether target or non-target) must have reduction in short axis to <10 mm.

Partial Response (PR): At least a 30% decrease in the sum of diameters of target lesions, taking as reference the baseline sum of diameters.

Progressive Disease (PD): At least a 20% increase in the sum of diameters of target lesions, taking as reference the smallest sum on study (this may include the baseline sum). The sum must also demonstrate an absolute increase of at least 5 mm.

Stable Disease (SD): Neither sufficient shrinkage to qualify for PR nor sufficient increase to qualify for PD.

Confirmation

In non-randomized trials where response is the primary endpoint, confirmation of PR and CR is required to ensure responses identified are not the result of measurement error. This will also permit appropriate interpretation of results in the context of historical data where response has traditionally required confirmation in such trials. However, in all other circumstances, (i.e., in randomized phase II or III trials or studies where stable disease or progression are the primary endpoints), confirmation of response is not required since it will not add value to the interpretation of trial results. However, elimination of the requirement for response confirmation may increase the importance of central review to protect against bias, in particular in studies which are not blinded. In the case of SD, measurements must have met the SD criteria at least once after study entry at a minimum interval (in general not less than 6–8 weeks) that is defined in the study protocol.

8.1 Primary Endpoint:

The proportion of patients on either arm achieving a PSA nadir of 4 or less (< 4 ng/ml) at month 7

of protocol therapy.

8.2 Response Rate

For measurable disease response, RECIST 1.1 criteria will be used. The Prostate Cancer Clinical Trials Working Group (PCWG2) criteria will be used to determine a PSA response [15]. PCWG2 recommends a two-objective paradigm in metastatic CRPC: (1) controlling, relieving, or eliminating disease manifestations that are present when treatment is initiated and (2) preventing or delaying disease manifestations expected to occur. PSA decline, changes in imaging will also be reported. The progression for measurable disease will be per RECIST1.1 criteria, and for bone metastases will be per PCWG2.

8.3 Duration of overall response

The duration of overall response is measured from the time measurement criteria are met for CR/PR (whichever is first recorded) until the first date that recurrent or progressive disease is objectively documented (taking as reference for progressive disease, the smallest measurements recorded since the treatment started).

8.4 Duration of stable disease

Stable disease is measured from the start of the treatment until the criteria for progression are met, taking as reference the smallest measurements recorded since the treatment started. Tumor evaluations are recommended every three cycles while on protocol therapy and then as clinically required by the treating physician.

8.5 Time to Treatment Failure:

From date of registration to date of progressive disease, or date patient is taken off study for any reason. All patients, regardless of the number of completed cycles of therapy, will be included for toxicity evaluation. All subjective toxicities encountered during the study will be evaluated according to the NCI toxicity criteria. Objective toxicities will be based upon the greatest severity that occurred at any time during a treatment cycle and not on the severity noted at the beginning of the next cycle.

8.6 Time to Progression:

From date of registration to date of progressive disease, defined as either progression of measurable disease by RECIST 1.1 criteria, or progression of bone metastases or occurrence of new skeletal related event or by PSA per PCWG 2 criteria [Appendix B].

8.7 Time to Progression in patients with bone metastases

Majority of the patients with prostate cancer have bone metastases which are not considered measurable sites of disease. Progression in patients with bone metastases will be defined as either the appearance of a minimum of 2 new lesions on bone scan which are related to metastatic disease per the judgement of the treating physician, or the occurrence of a new skeletal related event or by PSA criteria per PCWG 2. Skeletal-related event is defined as occurrence of new pathologic bone fractures (vertebral or nonvertebral), spinal cord compression, requirement of surgery or radiation therapy to bone metastases (including the use of radioisotopes), or a requirement to change antineoplastic therapy to treat bone pain or other related symptoms.

8.8 Progression Free Survival

Time from registration to PSA progression as defined by PCWG 2 criteria or measurable disease progression by RECIST1.1 or skeletal disease progression by appearance of new metastatic sites on bone scan or clinical progression as determined by treating physician assessment, or death from any cause, whichever occurs first.

8.9 Follow up /Overall survival

Overall survival will be measured from date of registration to death or last follow up.
After treatment is discontinued for any reason patients will be followed every 3 months for progression and survival.

8.10 Response Evaluable Patients

All patients registered on the protocol and completing seven months of androgen deprivation therapy and getting the month 6 and 7 PSA levels evaluated are considered evaluable.

8.11 Toxicity- Evaluable patients

All patients registered on the protocol and starting therapy will be considered toxicity evaluable. Toxicity will be graded per the CTCAE criteria 4.0.

8.12 Skeletal-related event

Defined as occurrence of new pathologic bone fractures (vertebral or nonvertebral), spinal cord compression, requirement of surgery or radiation therapy to bone metastases (including the use of radioisotopes), or a requirement to change antineoplastic therapy to treat bone pain or other related symptoms.

8.13 Circulating Tumor Cells Response

The outcome measures associated with this definition are the following:
Measurements of CTC counts pre-therapy, post-therapy and the change between time points.
To assess the pre-therapy circulating tumor cell (CTC) counts and the changes in CTC after 1 month of therapy, and to associate them with the primary endpoint. CTC counts will be measured at baseline (day 1), and month 1 (days 28-42) of therapy. The pre and post-therapy CTC counts ;<5/7.5 ml which is considered favorable, vs >5/7.5 ml which is considered unfavorable, have been shown to be prognostic in metastatic CRPC patients treated with cytotoxic chemotherapy.

8.14 Correlative tests:

Androgen metabolism enzymes: ERG, CXCR4, Androgen biosynthetic enzymes: Testing will be conducted on paraffin embedded fixed tissue. Analysis will be performed by Immunohistochemistry. Scoring will be between 0 to 3 and will be done by 2 independent experienced investigators.

Androgen biosynthetic enzymes: ERG, CXCR4 testing is conducted on RNA from frozen tumor tissue biopsies. RNA message measurement by qualitative and quantitative RT- PCR (real time PCR). Qualitative Scoring for RT-PCR is the presence or absence of band (+/-) when an

amplified PCR mixture is run on gel. Quantitative PCR is the level of expression of the test gene will be expressed in in terms of folds.

Genomic testing:
AR splice variants: Starting material is frozen tissue. Analysis is RNA message quantitation by RT-PCR and QPCR, which is similar to androgen biosynthesis analysis: Qualitative (+/-) and quantitative results (copy number) will be obtained.

9.0 CRITERIA FOR REMOVAL FROM STUDY:

- **9.1** Intercurrent illness which would, in the judgment of the investigator, a) affect assessments of clinical status to a significant degree, b) require discontinuation of drug, or c) both a and b.
- **9.2** Receiving any other treatment, investigational agent or alternative therapy directed at prostate cancer.
- **9.3** Unacceptable toxicity
- **9.4** Disease progression
- **9.5** Delay in treatment of $\geq$ 2 months
- **9.6** The patient may withdraw from the study at any time for any reason.
- **9.7** In the judgment of the investigator, further treatment would not be in the best interest of the patient.

10.0 CORRELATIVE STUDIES

10.1 <u>Bio-specimens</u>: Pretreatment tumor tissue biopsies will be mandatory for study enrollment. If patient has accessible bone metastases then bone biopsy is required. If patient has no bone metastases amenable to biopsy then tumor tissue biopsy can be conducted from other sites. Post treatment core biopsies of tumor tissues will be encouraged and obtained from the patients in both arms. Archival primary prostate tissue slides/blocks submission is required. A minimum of 10 unstained slides or block should be sent to Dr Chinni (see address below in section 10.7). **An additional optional tumor tissue biopsy will be requested when patients are progressing on study medication**. This phase constitutes castrate resistant, where patients were progressing on androgen ablation and androgen receptor targeted therapies. Collection of metastatic biopsy from the patients at this phase will be offered as an option. Molecular studies and data on the clinical samples at this stage are limited and we have a unique opportunity to collect the patient tissues at this phase. Genomic testing and evaluation of tumor tissue will be done by Caris Inc. and additional analysis will be done in Dr Chinni's lab. We are collecting tumor biopsy specimens prior to treatment and collection of specimens at the CRPC phase will improve our ability to monitor the biomarker response and determine pathways of resistance. The tissue procurement, handling and shipping procedures will be the same for each biopsy.

10.2 <u>Biomarkers:</u> CXCR4, ERG and key androgen biosynthesis enzymes (SRD5A2, CYP17, CYP3A4, AKR1C3 and HSD17b6) will be analyzed for mRNA and protein expression in bio specimens. AR CAG repeats and androgen receptor variant expression will be determined in tumor tissues.

10.3 <u>Background and rationale</u>: Prostate cancer cells over express CXCR4 receptor which has been critically involved in disease progression [16]. We showed that CXCR4 expression regulates key signaling pathways contributing to invasion of tumor cells [17] and bone tumor growth [18]. TMPRSS2-ERG fusions are frequently expressed in PC patients where androgens regulate ERG factor expression in PC cells [19]. We and others found a link between ERG and CXCR4 expression in prostate cancer patient tissues 20, 21].

CXCR4 and TMPRSS2-ERG fusion genes were shown to be differentially regulated in human tumor tissues in African American and Caucasian men. CXCR4 has been shown to be associated with aggressive tumor tissues of African American men, whereas TMPRSS2-ERG fusions were significantly localizes to prostate tumors in Caucasian men [22-24]. CXCR4 expression has been shown to be key molecular predictor of bone metastasis [16,19,25]. TMPRSS2-ERG fusion status is not known in bone metastasis. Published data demonstrate that TMPRSS2-ERG fusions are early markers for prostate cancer, but their expression is sustained in advanced cancers suggesting that fusion activated ERG factor is still functional in tumor cells [26]. Both CXCR4 and TMPRSS2-ERG are androgen responsive genes in prostate tumor cells [17,18]. Androgen biosynthesis enzyme expression has been shown to be persistent in advanced patients, who undergo hormonal therapy [27]. We will analyze the expression of key enzymes (SRD5A2, CYP17, CYP3A4, AKR1C3 and HSD17b6) contributing to intratumoral androgen biosynthesis. Pre and post treatment tumor tissues in both arms of the study will be analyzed for marker expression.

Trinucleotide CAG repeats in AR regulates AR signaling. AR with shorter repeats enhances the AR transcriptional activity compared to the longer repeats [28, 29]. Expression of shorter repeats in prostate tumors associates with high grade, PSA expression and high risk of biochemical failure [6]. Even though, the length of AR CAG repeat polymorphisms is not risk factor for prostate cancer incidence in different ethnic populations[,30, 31], shorter AR CAG repeats are highly prevalent in men with African American decent[, 32, 33]. The presence of shorter repeats in African American patients translates to stronger AR expression [34, 35] and its signaling leading to aggressive disease characteristics. In the present study we will evaluate AR CAG repeat lengths in patient tumor tissues and determine the effect of AR CAG repeat lengths on the treatment responsiveness.

Androgen receptor variants lacking ligand binding domain were recently identified in castrate resistant prostate cancer patient tumors through deep sequencing of prostate tumor tissues[36]. Presence of these variants confer the ligand independent activation of AR thus lead to resistant to common AR targeted therapies which commonly attack the AR at ligand binding domain. Even though, there are several types of AR variants identified; their importance in prostate cancer progression is currently being actively investigated. A recent study further shows that expression of AR variant (AR-V7) in hormone naïve patients associated with earlier disease relapse in prostatectomy patients [37]. In CRPC patients expression of AR variants inversely correlate with survival [34]. Preclinical studies show that expression of AR variants confer aberrant androgen independent growth of prostate cancer cells [38]. In addition, AR variant expression confers resistance to anti-AR agents. The prevalence of these AR variants` expression in tumor tissues is an active area of investigation to determine the importance of AR variant expression on clinical progression of prostate cancer. In the present study we will evaluate the expression of AR variants AR-V1, AR-V6, AR-V7 and AR-v567es expression in pretreatment and post treatment groups [39]. Expression of these variants will be evaluated for therapy response.

10.4 Tissue procurement: Prostate tumor biopsy specimens will be cut into three pieces, two pieces will be snap frozen in liquid nitrogen for RNA and DNA extraction and other will be placed in 10% phosphate buffered formalin. For metastatic tumor biopsies we prefer five core biopsies that will be collected for analysis of which two cores will be directly placed into 10 % phosphate buffered formalin and processed for decalcification and histological analyses. Remaining three core biopsies will be immediately snap-frozen in liquid nitrogen and transported to Dr. Chinni's laboratory, where they will be stored in a -80ºC freezer until ready for use. The contact person for tissue collection is Dr. S.R. Chinni at 313 577 1533.

The technician performing the collection will write on a note sheet any information on biopsy performance, including comments regarding core quality (long cylindrical core, bone shards, mostly blood clot, etc), the quantity of cores, and any irregularities in the freezing process, along with the technician's name, the biopsy date, and the time of the procedure. Frozen biopsies coming from lesions with more than 60% tumor involvement, as confirmed by review of the H&E paraffin-embedded frozen section, will be selected for DNA and RNA extraction and used for gene expression and AR CAG repeat measurements and future molecular analysis.

10.5 Methods:

Immunohistochemistry: Formalin fixation will be performed for 24 hours followed by routine procedure of dehydration, paraffin embedding and preparation of tissue sections. For bone biopsy specimens decalcification will be performed in EDTA solution prior to dehydration and paraffin embedding. Histological analyses of the tissue sections will be performed with hematoxylin and eosin staining and immunohistochemical analysis will be performed for CXCR4, ERG and androgen biosynthesis enzymes (SRD5A2, CYP17, CYP3A4, AKR1C3 and HSD17b6).

Gene expression analysis: Frozen tissues will be homogenized and total RNA will be extracted using Trizol method. cDNA will be synthesized from RNA and RT-PCR analysis will be performed with gene specific primers to determine the expression of genes in biopsy samples. Real Time PCR will be performed with cDNA, gene specific primers and reaction mixture in Eppendorf PCR machine. CXCR4, ERG, androgen biosynthesis enzymes (SRD5A2, CYP17, CYP3A4, AKR1C3 and HSD17b6) and AR variants (AR-V1, AR-V6, AR-V7 and AR-v567es) primers will be used in RT-PCR and amplified band will be analyzed on 0.7 % acrylamide gel for detecting presence or absence of gene expression. The gene expression will be quantitated u using the $2^{-\Delta\Delta Ct}$ method, where $\Delta\Delta Ct = (\Delta Ct_{target\ gene} - \Delta Ct_{GAPDH})_{sample} - (\Delta Ct_{target\ gene} - \Delta Ct_{GAPDH})_{control}$. A prostate cancer cell line, VCaP expresses ERG, CXCR4, androgen biosynthetic enzymes and AR variants and will be used as a positive control in the experiment.

DNA Isolation: DNA will be isolated from frozen tissue using a Qiagen EZ1 Advanced using magnetic bead technology with the "Isolation of DNA from Soft Tissue using the TissueLyser" user-developed protocol, the EZ1 DNA Tissue Card, and the EZ1 DNA Tissue Kit. Purified DNA will be quantified by UV spectrophotometry using a DropSense96® Microplate Spectrophotometer (Trinean) and the purity assessed based on the A260/A280 and A260/A230 ratios. A260/A280 ratio of 1.8 is considered good quality DNA.

Genotyping: The Androgen Receptor CAG length polymorphism will be interrogated by PCR amplification carried out in a 15 µl reaction containing 20 µmol/l of the gene specific primers NED-gcgcgaagtgatccagaa and gttgctgttcctcatcca (Rosa et al, 2006), 1X True Allele PCR Mix (Applied Biosystems), and 25 ng genomic DNA. The amplification will be performed on a Mastercycler Gradient thermocycler (Eppendorf) with reaction conditions 95^0C for 10 minutes, 40 cycles of amplification of 95^0C for 30 seconds, 60.6^0C for 30 seconds, and 72^0C for 30 seconds followed by a final extension of 72^0C for 10 minutes. The NED-labeled PCR products will be mixed with LIZ-labelled GeneScan™ 500 size standard (Applied Biosystems) and electrophoresed on a QuantStudio 12K Flex (Life Technologies). Two DNA standards have been established in the Wayne State University Applied Genomics Technology Center by Sanger sequencing (AB 3730) for 18 (CEPH 1347-02, Life Technologies) and 21 CAG repeats. These samples will be run on each 96-well plate as positive controls. No template controls will also be run. The data will be analyzed with GeneMapper (Life Technologies).

DNA Sequencing: DNA sequencing reactions will be carried out using the 0.5X protocol for ABI PRISM BigDye Terminator Cycle Sequencing Ready Reaction Kit (Applied Biosystems). The sequencing extension products will be purified utilizing Sephadex. The purified products will be analyzed on an ABI PRISM 3730 DNA Analyzer using a 50 cm capillary array.

10.6 Packaging instructions:

The bone tumor core biopsies will be directly ejected into 10 % formalin PBS and should be sent directly to Dr. S.R. Chinni. Technician collecting the core biopsies should write study ID number of the patient and the time and date of collection. The tubes should be wrapped with a bubble wrap to prevent accidental breakage of tube during shipping. The tubes containing core biopsies in 10 % formalin PBS should be sent to Dr. SR. Chinni, in a FedEx envelope in a next day delivery. An email should be sent toschinni@med.wayne.edu about the delivery of FedEx shipment.

Formalin preserved biopsies of bone tumor cores will be processed in Dr. Chinni's lab

10.7 Shipping instructions for frozen tumor biopsies:

The core should be directly dropped into an empty sterile tube. The tube containing core biopsy should be placed immediately on dry ice. The dry ice package should be sent on that day (cannot be batched) to Dr. SR. Chinni for next day delivery.

Any deviations in collection procedure should be noted and information should be sent to Dr. SR. Chinni schinni@med.wayne.edu.

Please email Dr Chinni and the data manager Kimberlee Dobson (dobsonk@karmanos.org or Tel no 313-576-9837) or a covering data manager regarding the samples that will be sent, with a copy of the specimen worksheet that will be mailed with the specimen

A specimen collection worksheet must accompany each shipment. All samples (tumor tissue biopsies and archival tissue slides/block) should be clearly labeled with study site, patient ID number, date of collection (for fresh biopsies) and then sent to:

Sreenivasa R. Chinni, Ph.D.
Departments of Urology, Oncology and Pathology,
KCI, Wayne State University School of Medicine,

9245, Scott Hall
540 E Canfield Ave
Detroit, MI 48201.
Tel # 313-577-1833
E mail : schinni@med.wayne.edu

Signaling via steroid receptors including androgen receptor (AR) requires sustained ligand synthesis. The hydroxysteroid dehydrogenase (HSD) family of enzymes (such as HSD3B1) are critical for AR signaling and the development of CRPC, and likely the development of resistance to next generation hormonal therapies including enzalutamide. We plan to interrogate expression of HSD family enzymes pre-enzalutamide treatment, in treatment and with the development of resistance. We suspect that expression of some of these enzymes required for ligand synthesis will increase with resistance. One paraffin embedded slide from four patients which have both pre and post met biopsy will be sent for this testing. Additional samples maybe sent based on availability.

Please contact Marianne Petro Tel no 216 445-9751, E mail petrom@ccf.org prior to sending specimen.
The slides will be sent (depending on tissue availability) to:
Marianne Petro, Sharifi lab
Cancer Biology, NB4-137
Lerner Research Institute
Cleveland Clinic Foundation
2111 East 96th Street
Cleveland, OH 44106

Additional testing is proposed on metastatic biopsies collected in this study. Fluorescence in situ hybridization will be conducted to determine the gene fusions in biopsy specimens. This assay will be performed by Dr. Nallasivam Palanisamy at Henry Ford Health Systems. One paraffin embedded tissue slide will be given to Dr. Palanisamy and he will utilize fluorescent probes to determine the gene fusions.
Further analysis proposed includes metastatic tissue RNA samples and paraffin embedded slides for gene expression studies relevant to prostate cancer growth, metastasis and therapy resistance. This testing will be performed in Dr. Chinni laboratory.

10.8 Circulating Tumor Cells (CTC)

In metastatic CRPC, CTC are gaining importance as a method to assess response. In a disease where the sites of metastases are mainly in bone which is not measurable disease the CTC counts are a critically important less invasive method of predicting response and outcome. **CTC counts will be measured at baseline (day 1), and month 1 (days 28-42) of therapy.** The pre and post-therapy CTC counts ;<5/7.5 ml which is considered favorable, vs ≥5/7.5 ml which is considered unfavorable, have been shown to be prognostic in metastatic CRPC patients treated with cytotoxic chemotherapy [*40*]. In multivariable analysis, CTC count using Veridex technology was an independent risk factor, influencing overall survival (OS) in a multivariable analysis. The differences in survival associated with CTC count status were clinically and statistically significant (e.g., ~1 year, depending on the comparison). Investigators are now exploring the role of CTC counts as surrogate markers of survival in CRPC, especially when used in conjunction with cytotoxic chemotherapy.

The CTC counts will be performed by the Correlative Sciences Lab at Karmanos cancer Institute.
Cell save tube (8ml) for CTC to be collected at baseline, and after 28-42 days of therapy.
Cell Save Tubes will be provided and should be labeled after collection with study site, patient study ID and date and time of collection and shipped within 24 hours **(cannot be batched)** to:
A specimen collection worksheet must accompany each shipment. CTC and tumor biopsy specimens should only be collected on Mon, Tue or Wed of any week for participating sites other than those within Detroit area to allow for prompt shipping and delivery.

Attn: Karri Stark PhD
Barbara Ann Karmanos Cancer Institute
Hudson-Webber Cancer Research Center,
Correlative Sciences Lab Rm 808
4100 John R. Street
Detroit, Michigan 48201
Tel.: 313-576-8248 (lab)
E mail: starkka@karmanos.orgEmail:wuji@karmanos.org

Please email Dr Stark and the data manager Kimberlee Dobson (dobsonk@karmanos.org or Tel no 313-576-9837) or a covering data manager regarding the samples that will be sent, with a copy of the specimen worksheet that will be mailed with the specimen.

11.0 STATISTICAL CONSIDERATIONS:

11.1 Primary objective:

To compare the rates of achieving PSA remission at month 7 with LHRH analogue therapy and enzalutamide (Arm A) and compare with that achieved with LHRH analogue and bicalutamide (Arm B) in metastatic hormone sensitive prostate cancer.

11.2 Secondary Objectives:

1) To compare the primary endpoint by race (African American [AA] vs Other).

2) To compare the rates of each of 2 types of response by treatment arm:
   a) Measurable disease response; and
   b) PSA response.

3) To compare each of 7 time-to-event endpoints by treatment arm:
   a) Duration of overall response (RD);
   b) Duration of stable disease (SDD);
   c) Time to treatment failure (TTF);
   d) time-to-progression (TTP);
   e) TTP in patients with bone metastases;
   f) progression-free survival (PFS); and
   g) Overall survival (OS).

4) To compare the rates of each type of toxicity by treatment arm.

5) To compare the incidence rate of skeletal related events (SRE), and the time until SRE, separately by treatment arm.

6) To compare the rates of CTC response by treatment arm..

7) To explore the molecular mechanisms within the androgen receptor pathway by determining the levels of CXCR4 and TMPRSS2-ERG expression, androgen metabolism enzymes, androgen receptor variants, and length of CAG repeats within the androgen receptor gene, and to associate them with the primary endpoint.

11.3 Endpoints

11.3.1 For the primary objective, the binary endpoint will be the achievement of PSA remission at month 7 (yes or no). This endpoint is described in the Study Schema and in Section 8.1.

11.3.2 For secondary objective (1), the primary endpoint is described in the Study Schema and in Section 8.1. The two race groups to be compared are AA vs Other, as described in the Study Schema.

11.3.3 For secondary objective (2), the endpoints will be the achievement of:

a) measureable disease response (yes or no), (defined in Sections 8.0 and 8.9); and
b) PSA response (as defined in Section 8.2).

11.3.4 For secondary objective (3), the endpoints will be the length of censored:

a) duration of overall response (RD), as defined in Section 8.3;
b) duration of stable disease (SDD), as defined in Section 8.4;
c) time to treatment failure (TTF), as defined in Section 8.5;
h) time-to-progression (TTP), as defined in Section 8.6;
i) TTP in patients with bone metastases, as defined in Section 8.7;
j) progression-free survival (PFS), as defined in Section 8.8; and
k) overall survival (OS), as defined in Section 8.9.

11.3.5 For secondary objective (4), the endpoints will be the occurrence rate of each specific type of toxicity observed (defined in Section 6.0) and treatment arm (A or B, defined in Study Schema, Section 5.3, and Section 11.1).

11.3.7 For secondary objective (5), the endpoints will be the occurrence rate of SRE (defined in Section 8.12), the distribution of time until SRE, and treatment arm (A or B). Follow-up for SRE will continue until SRE, PSA progression, or death, whichever occurs first.

11.3.8 For secondary objective (6), the endpoints will be the occurrence rate of CTC response (defined in Section 8.13), and treatment arm (A or B).

11.3.9 For secondary objective (7), the endpoints will be the expression levels of the selected genes or enzymes stated in Section 11.2, length of CAG repeats, and the primary endpoint of PSA remission at month 7 (yes or no). The correlatives are defined and described in Section 10.0. The expression level of those biomarkers will be determined in both pre- and post-treatment prostate tumor tissues, and in post-treatment bone biopsy samples

11.4 Design, Stratification, and Randomization

11.4.1 Design

This trial will use a randomized Phase 2 *screening* design (RP2SD) as described by Rubinstein et al [41]. The RP2SD permits *a non-definitive comparison* of the primary outcome by treatment. With its associated relaxed alpha and/or beta, we are looking for a clinically relevant signal of efficacy differential in the primary endpoint, to determine whether to plan a larger subsequent study.

11.4.2 Stratification and Randomization

Patients will be stratified by race (AA or other) and bone pain (present or absent) into one of 4 strata. A separate randomization list for each stratum will prepared in advance by the study biostatistician. Patients will then be randomized to Arm A or Arm B using the stratum-appropriate randomization list. Each of the 4 randomization lists (sequences) will consist of

random permuted blocks of varying sizes.

11.5 Sample Size Determination

The assumptions made to permit sample size determination are:

1) The population reference value of the proportion of patients achieving (and then sustaining) PSA ≤ 4 ng/ml (4 or less) within 7 months of starting therapy is 50% for men on the standard (i.e., control arm, or Arm B) therapy of LHRH agonist + bicalutamide;

2) The hypothesized rate of patients achieving (and then sustaining) PSA ≤ 4.0 within 7 months of starting therapy on the experimental arm (i.e., enzalutamide, or Arm A) is 75%;

3) significance level of 0.10, one-sided (in view of the directional hypothesis);

4) power = 0.80;

5) balanced (i.e., 1:1) randomization.

11.5.1 Under those assumptions, the required sample size is N=41 response-evaluable (r-e) patients (defined in Section 8.10) on each arm. That calculation used the method of Fleiss et al [42], and was performed with the nQuery Advisor software, Version 7.0. The total sample size required is 82 patients. Allowing for up to 10% of patients who might not become r-e, up to 46 patients per arm (a total of 92 patients) may need to be enrolled.

11.5.2 Enrollment to this study is expected to be approximately 25-30% AA (from all participating institutions). For better generalization of our study results, we seek a minimum accrual of 25 (approximately 30%) evaluable AA patients. To achieve that goal, it may become necessary to close accrual to non-AA patients at some point during the study. Let p = the primary endpoint (the proportion of patients achieving PSA ≤ 4.0 within 7 months). With 25 AA patients, p can be estimated to within 0.183 of its true value with 95% confidence, regardless of the level of p.

11.6 Analysis

11.6.1 For the primary objective, the binary endpoint (PSA remission at month 7, yes/no) will be summarized with its point estimate (an occurrence rate), and 2-sided Wilson type 95% confidence interval (CI). This analysis will be performed two ways: a) for all randomized patients (the intention to treat subset); and b) for all r-e patients (the per protocol subset). PSA response rates will be compared by treatment arm in a stratified logistic regression model, with stratification by race (AA, Other) and bone pain (yes, no).

11.6.2 All binary endpoints in secondary objectives 1-2 and 4-7, will be summarized with point estimates (occurrence rates), and 2-sided Wilson type 95% CIs. The analysis of toxicity rates will be performed using only the toxicity-evaluable (t-e) patients (defined in Section 8.11).

11.6.3 For secondary objective 4, the grade of each type of toxicity (an ordinal categorical variable) will be summarized with 1-way frequency distributions. A similar descriptive analysis will be performed for any correlatives in secondary objective 7 to be assayed by IHC, resulting in ordinal categorical variables (intensity levels of 0, 1+, 2+, or 3+).

Dichotomized versions (e.g., the occurrence rate of Grade 3-4 neutropenia) will be summarized as described in Section 11.6.2. A similar descriptive analysis will be performed for the presence/absence of any correlatives in secondary objective 7 to be assayed by IHC, with presence defined as intensity level of 1+ through 3+.

For any correlatives in secondary objective 7 that are assayed in both pre- and post-treatment prostate tumor tissues, the change in IHC expression level will be described using a 2-way frequency distribution. If the effective sample size appears to be sufficient relative to the size of the 2-way table, the association of these paired variables can be tested with the exact version of McNemar's test.

11.6.4 All censored time-to-event distributions for secondary objectives 3 and 5 will be estimated with standard Kaplan-Meier (K-M) methodology. Point and CI estimates of the median and various time point-specific rates will be derived from the K-M life table. The censored K-M distributions will be compared with the log-rank test, stratified by race (AA, Other) and bone pain (yes, no).

11.6.5 All (uncensored) continuous variables from secondary objective 7 (e.g., ERG or CXCR4 or AR splice variant level measured by QPCR in frozen tissue, or length of CAG repeats) will be summarized with descriptive statistics including the point estimate of the mean, its associated 95% CI (or 90% CI, depending on N), median, IQR, standard deviation, minimum, and maximum. Gene expression levels reported in terms of the number of fold increases will be log_2-transformed before summary statistics are calculated.

If such variables are pseudo-continuous (e.g., 10-15 unique values), they will also be analyzed as categorical variables using 1-way frequency distributions, as described in Section 11.6.3.

Pre- and post-treatment CTC counts (required to determine CTC response for secondary objective 6), will be similarly analyzed as continuous variables. Depending on their distribution, CTC counts will also be analyzed as categorical variables in the manner described in Section 11.6.5 for length of CAG repeats.

11.6.6 The association of correlative markers (whether binary, or ordinal categorical, or continuous) with a binary clinical outcome (e.g., the primary endpoint of achievement of PSA remission at month 7, yes/no) will be explored using exact logistic regression models. Point and CI estimates of the odds ratio (OR) will be calculated, but the statistical interpretation will be limited to only the direction, magnitude, and precision of the association (i.e., the OR) as these are only exploratory analyses.

11.6.7 The association of correlative markers (whether binary, or ordinal categorical, or continuous) with a time-to-event clinical outcome (e.g., OS, PFS, TTP, or time to SRE) will be explored using Cox proportional hazards (PH) regression models. Point and CI estimates of the hazard ratio (HR) will be calculated, but the statistical interpretation will be limited to only the direction, magnitude, and precision of the association (i.e., the HR) as these are only exploratory analyses.

11.7 Accrual rate, accrual duration, and study duration

11.7.1 Six institutions are expected to enroll patients onto this study: KCI, Henry Ford Hospital, University of Alabama at Birmingham, Ohio State University (OSU), Mount Sinai School of Medicine, and the Cancer Institute of New Jersey (CINJ). Their accrual rates are expected to be similar, at about 5-7 patients/year.

11.7.2 That yields a combined 6-institution expected accrual rate of 30-42 patients/year. At that expected accrual rate, it should take 27-37 months to enroll 92 patients (46 per treatment arm) among whom we should have at least 82 (41 per treatment arm) who are r-e for the primary endpoint.

11.7.3 Since a follow-up period of up to 7 months is required to determine the primary endpoint of the study, then the expected total study duration is 34-44 months.

12.0 ADVERSE EVENTS REPORTING

12.1 Regulatory and reporting requirements- These will be followed per IND requirements (if applicable) as well as sponsor requirements and WSU IRB requirements

12.2 Definition of Adverse Events (AE)

An adverse event (AE) is defined as any untoward medical occurrence in a subject administered a study drug and which does not necessarily have a causal relationship with this treatment. An AE can therefore be any unfavorable and unintended sign (including an abnormal laboratory finding), symptom, or disease temporally associated with the use of a study drug, whether or not related to the study **OR**

An abnormality identified during a medical test (e.g., laboratory parameter, vital sign, ECG data, physical exam) should be defined as an AE only if the abnormality meets one of the following criteria:

- induces clinical signs or symptoms.
- requires active intervention.
- requires interruption or discontinuation of study medication.
- the abnormality or investigational value is clinically significant in the opinion of the investigator.

12.3 <u>WSU IRB Adverse Event Reporting Guidelines</u>

Unexpected Problem – Risk to participant or others. A problem that is a) unforeseen b) indicates that participants or others are at increased risk of harm, and c) is related or possibly related to the research. The following are examples of unexpected problems:

1. <u>Adverse Event:</u> Any harm experienced by a participant regardless of whether the event was internal (on-site) or external (off-site) and regardless of whether the event meets the

FDA definition of “serious adverse event”, which in the opinion of the principal investigator are both **unexpected, related (definitely, probably, or more likely than not)**, **and suggests that participants are at greater risk than was previously known or recognized.**

a. An adverse event is “unexpected” when its specificity and severity are not accurately reflected in the informed consent document, the protocol or the investigator’s brochure.
b. An adverse event is “related to the research procedures” if in the opinion of the principal investigator it was more likely than not to be caused by the research procedures, or if it is more likely than not that the event affects the rights and welfare of current participants.

2. **Any harm experienced by a participant or others** as a result of involvement in research activities (internal or external **excluding** adverse events).
3. Information that indicates a **change to the risks or potential benefits** of the research. For example:
   a. An interim analysis or safety monitoring report indicates that frequency or magnitude of harm or benefit may be different than initially presented to the IRB.
   b. A paper is published from another study that shows the risks or potential benefits to your research may be different than initially presented to the IRB.
   c. Study put on hold by the PI, FDA, or the Sponsor for reasons that may include safety, toxicity and/or efficacy.
4. **A change in FDA labeling** or withdrawal from marketing of a drug, device, or biologic used in a research protocol.
5. **Change to the protocol** taken without prior IRB review to eliminate an apparent immediate hazard to a research participant.
6. **Research conducted without prior WSU IRB approval**.
7. **Event that requires prompt reporting to the sponsor**.
8. **Unanticipated adverse device effect**: Any serious adverse effect on health or safety, or any life-threatening problem or death caused by, or associated with, a device if that effect, problem, or death was not previously identified in natured, severity, or degree of incidence in the investigational plan or application (including a supplementary plan or application), or any other unanticipated serious problem associated with a device that relates to the rights, safety, or welfare of subjects.
9. **Sponsor-imposed suspension for risk**.
10. **Complaint of a participant** when the complaint indicates unexpected risks or cannot be resolved by the research team.
11. **A breach of confidentiality**.
12. **Protocol violation/deviation** (meaning an accidental or unintentional change to the IRB-approved protocol) that harmed participants or others, that indicates participants or others may have been placed at increased risk of harm, or rights of research participants were violated. For example:
    a. Failure to draw safety labs
    b. Request for continuation submitted late to the IRB three years in a row
    c. Wrong informed consent signed or failure to obtain informed consent
13. **Incarceration of a participant** in a protocol not approved to enroll prisoners.

14. All deaths that have happened at WSU or one of its affiliates within 30 days of the last study intervention, and not related to progressive disease.

15. **Any death, if the PI feels that it is significant** no matter when it occurs.

IRB Policy

Principal investigators must report any of the above to the IRB as soon as possible, **but in all cases within 5 working days**.

12.4 Definition of Serious Adverse Events (SAES)

An AE is considered “serious” if, in the view of either the investigator or sponsor, it results in any of the following outcomes:

- results in death,
- is life threatening (an ae is considered “life-threatening” if, in the view of either the investigator or sponsor, its occurrence places the subject at immediate risk of death. it does not include an AE that, had it occurred in a more severe form, might have caused death.),
- results in persistent or significant disability/incapacity or substantial disruption of the ability to conduct normal life functions,
- results in congenital anomaly, or birth defect,
- requires inpatient hospitalization or leads to prolongation of hospitalization (hospitalization for treatment/observation/examination caused by ae is to be considered as serious),
- other medically important events.

12.5 Serious Adverse Event Reporting (SAE)

The SAE reporting will begin at the time of registration until 30 days after the last dose of either Enzalutamide or Bicalutamide. Within 24 hours of awareness of a serious adverse event, whether or not related to the study drug, the investigator will complete and submit a Medwatch 3500a form, containing all required information (reference 21 CFR 312.32). The investigator will submit a copy of this Medwatch 3500a form to the coordinating institution (PI- Dr Heath) and Astellas by either e-mail or fax, within the same timeframe. Reporting to Astellas is only required for SAEs occurring on patients randomized to Arm A.

The SAE documentation, including the Medwatch 3500A form and available source records should be emailed or faxed to:

Astellas pharma global development – United States

Email: safety-us@us.astellas.com

Fax number: (847) 317-1241

The following minimum information is required:

- study number/IIT regulatory identifier
- subject number, sex and age
- the date of report
- a description of the SAE (event, seriousness of the event)
- causal relationship to the study drug. Follow-up information for the event should be sent within 7 days as necessary. Copy of above should also be sent to coordinating site (PI- Dr Heath).

Study Coordination Center/Principal Investigator:
Elisabeth Heath, MD..D.
Contact Information 4HWCRC, 4100 John R
Detroit MI 48201.
Tel# 313-576-8717 and fax # 313-576-8767
E mail: heathe@karmanos.org

Every institution should also report SAE to their respective IRB per institutional guidelines.

12.6 Procedure in case of Pregnancy

The effect of enzalutamide in pregnant and lactating women is not known, and the exposure of a fetus or nursing infant is considered a potential risk. enzalutamide can cause fetal harm when administered to a pregnant woman based on its mechanism of action. Subjects receiving enzalutamide are advised to use 2 acceptable methods of birth control (one of which must include a condom as a barrier method of contraception) starting at the time of screening for an enzalutamide study and continuing throughout the course of treatment and for at least three months after enzalutamide is discontinued.

If during the conduct of the clinical trial, a male subject impregnates his partner, the subject should report the pregnancy to the investigator. the investigator should report the pregnancy to the sponsor as an SAEwithin 24 hours of awareness of the event. The expected date of delivery or expected date of the end of the pregnancy, last menstruation, estimated fertility date, pregnancy result and neonatal data etc., should be included in this information.

The investigator should report the outcome of the pregnancy (independent of outcome, eg. full term delivery, pre-term delivery, spontaneous abortion, induced abortion, stillbirth, death of newborn, congenital anomaly [including anomaly in a miscarried fetus, etc] in accordance with the same reporting procedure as for SAEs. The date of outcome of the pregnancy, gestational age, date of birth and neonatal data etc., should be included in this information.

12.7 <u>Protocol amendments, or changes in study conduct</u>

Any change or addition (excluding administrative) to this protocol requires a written protocol amendment that must be reviewed and approved. Amendments significantly affecting the safety of subjects, the scope of the investigation or the scientific quality of the study require additional approval by the IRB at each study center. A copy of the written approval of the IRB must be provided to. Examples of amendments requiring such approval are:

1. increases in drug dose or duration of exposure of subjects,
2. significant changes in the study design (e.g. addition or deletion of a control group),
3. increases in the number of invasive procedures,
4. addition or deletions of a test procedure required for monitoring of safety.

These requirements for approval should in no way prevent any immediate action from being taken by the investigator or by in the interests of preserving the safety of all patients included in the trial. If an immediate change to the protocol is felt to be necessary by the investigator and is implemented for safety reasons PI must be notified and the IRB at the center must be informed immediately. Amendments affecting only administrative aspects of the study do not require formal protocol amendments or IRB approval but the IRB must be kept informed of such

administrative changes. Examples of administrative changes not requiring formal protocol amendments and IRB approval include:

1. changes in the staff used to monitor trials
2. minor changes in the packaging or labeling of study drug

13.0 References:

1. Hussain M, Tangen CM, Higano C, et al. Absolute prostate-specific antigen value after androgen deprivation is a strong independent predictor of survival in new metastatic prostate cancer: data from Southwest Oncology Group Trial 9346 (INT-0162). J Clin Oncol. 2006 Aug 20;24(24):3984-90.
2. Hussain M, Tangen CM, Berry DL et al. N Engl J Med 2013 Intermittent versus continuous androgen deprivation in prostate cancer. N Engl J Med. 2013 Apr 4;368(14):1314-25.
3. Tran C, Ouk S, Clegg NJ et al. Development of a second-generation antiandrogen for treatment of advanced prostate cancer. Science 2009; 324(5928):787-790.
4. AR Pienta KJ, Bradley D. Mechanisms underlying the development of androgen independent prostate cancer. Clin Cancer Res 2006; 12(6):1665-1671.
5. Scher HI, Fizazi K, Saad F, et al. Increased survival with enzalutamide in prostate cancer after chemotherapy. N Engl J Med. 2012 Sep 27;367(13):1187-97
6. Scher HI, Beer TM, Higano CS et al. Antitumour activity of MDV3100 in castration resistant prostate cancer: a phase 1-2 study. Lancet 2010; 375(9724):1437-1446.
7. I. Al-Howaidi, L. Shatat, T. Tashi, W, et al. GU ASCO 2011 abstract 199.
8. Thatai LC, Banerjee M, Lai Z, Vaishampayan U. Racial disparity in clinical course and outcome of metastatic androgen-independent prostate cancer. Urology. 2004 Oct;64(4):738-43.
9. Thompson I, Tangen C, Tolcher A, et al. Association of African-American ethnic background with survival in men with metastatic prostate cancer.J Natl Cancer Inst. 2001 Feb 7;93(3):219-25.
10. Roach M 3rd, Lu J, Pilepich MV, Asbell SO, et al. Race and survival of men treated for prostate cancer on radiation therapy oncology group phase III randomized trials. J Urol. 2003 Jan;169(1):245-50.
11. Bennett CL, Price DK, Kim S,et al. Racial variation in CAG repeat lengths within the androgen receptor gene among prostate cancer patients of lower socioeconomic status. J Clin Oncol. 2002 Sep 1;20(17):3599-604.
12. Hatcher D, Daniels G, Osman I, Lee P. Molecular mechanisms involving prostate cancer racial disparity. Am J Transl Res. 2009 Apr 20;1(3):235-48.
13. Sweeney C, Chen YH, Carducci mA, et al. Impact on overall survival (OS) with chemohormonal therapy versus hormonal therapy for hormone-sensitive newly metastatic prostate cancer (mPrCa): An ECOG-led phase III randomized trial. J Clin Oncol 32;5S LBA2, 2014..
14. Eisenhauer A, Therasse P, Bogaert J, et al. New response evaluation criteria in solid tumors. Eur J Cancer 2009, 45: 228-47.

15. Scher H, Halabi S, Tannock I, et al. Design and End Points of Clinical Trials for Patients with Progressive Prostate Cancer and Castrate Levels of Testosterone: Recommendations of the Prostate Clinical Trials Working Group. J Clin Oncol 26(7):1148-59, 2008.

16. Sun YX, Wang J, Shelburne CE, Lopatin DE, Chinnaiyan AM, Rubin MA, Pienta KJ, Taichman RS: Expression of CXCR4 and CXCL12 (SDF-1) in human prostate cancers (PCa) in vivo. J Cell Biochem 2003, 89(3):462-473.

17. Chinni SR, Sivalogan S, Dong Z, Filho JC, Deng X, Bonfil RD, Cher ML: CXCL12/CXCR4 signaling activates Akt-1 and MMP-9 expression in prostate cancer cells: The role of bone microenvironment-associated CXCL12. The Prostate 2006, 66(1):32-48.

18. Chinni SR, Yamamoto H, Dong Z, Sabbota A, Bonfil RD, Cher ML: CXCL12/CXCR4 Transactivates HER2 in Lipid Rafts of Prostate Cancer Cells and Promotes Growth of Metastatic Deposits in Bone. Mol Cancer Res 2008, 6(3):446-457.

19. Tomlins SA, Rhodes DR, Perner S, Dhanasekaran SM, Mehra R, Sun XW, Varambally S, Cao X, Tchinda J, Kuefer R et al: Recurrent fusion of TMPRSS2 and ETS transcription factor genes in prostate cancer. Science 2005, 310(5748):644-648.

20. Cai J, Kandagatla P, Singareddy R, Kropinski A, Sheng S, Cher ML, Chinni SR: Androgens Induce Functional CXCR4 through ERG Factor Expression in TMPRSS2-ERG Fusion-Positive Prostate Cancer Cells. Translational oncology 2010, 3(3):195-203.

21. King JC, Xu J, Wongvipat J, Hieronymus H, Carver BS, Leung DH, Taylor BS, Sander C, Cardiff RD, Couto SS et al: Cooperativity of TMPRSS2-ERG with PI3-kinase pathway activation in prostate oncogenesis. Nat Genet 2009, 41(5):524-526.

22. Wallace TA, Prueitt RL, Yi M, Howe TM, Gillespie JW, Yfantis HG, Stephens RM, Caporaso NE, Loffredo CA, Ambs S: Tumor immunobiological differences in prostate cancer between African-American and European-American men. Cancer research 2008, 68(3):927-936.

23. Rosen P, Pfister D, Young D, Petrovics G, Chen Y, Cullen J, Bohm D, Perner S, Dobi A, McLeod DG et al: Differences in frequency of ERG oncoprotein expression between index tumors of Caucasian and African American patients with prostate cancer. Urology 2012, 80(4):749-753.

24. Magi-Galluzzi C, Tsusuki T, Elson P, Simmerman K, LaFargue C, Esgueva R, Klein E, Rubin MA, Zhou M: TMPRSS2-ERG gene fusion prevalence and class are significantly different in prostate cancer of Caucasian, African-American and Japanese patients. The Prostate 2011, 71(5):489-497.

25. Mochizuki H, Matsubara A, Teishima J, Mutaguchi K, Yasumoto H, Dahiya R, Usui T, Kamiya K: Interaction of ligand-receptor system between stromal-cell-derived factor-1 and CXC chemokine receptor 4 in human prostate cancer: a possible predictor of metastasis. Biochem Biophys Res Commun 2004, 320(3):656-663.

26. Cai C, Wang H, Xu Y, Chen S, Balk SP: Reactivation of androgen receptor-regulated TMPRSS2:ERG gene expression in castration-resistant prostate cancer. Cancer research 2009, 69(15):6027-6032.

27. Stanbrough M, Bubley GJ, Ross K, Golub TR, Rubin MA, Penning TM, Febbo PG, Balk SP: Increased expression of genes converting adrenal androgens to testosterone in androgen-independent prostate cancer. Cancer research 2006, 66(5):2815-2825.

28. Ding D, Xu L, Menon M, Reddy GP, Barrack ER: Effect of a short CAG (glutamine) repeat on human androgen receptor function. The Prostate 2004, 58(1):23-32.

29. Strom SS, Gu Y, Zhang H, Troncoso P, Babaian RJ, Pettaway CA, Shete S, Spitz MR, Logothetis CJ: Androgen receptor polymorphisms and risk of biochemical failure among prostatectomy patients. The Prostate 2004, 60(4):343-351.

30. Zeegers MP, Kiemeney LA, Nieder AM, Ostrer H: How strong is the association between CAG and GGN repeat length polymorphisms in the androgen receptor gene and prostate cancer risk? Cancer epidemiology, biomarkers & prevention : a publication of the American Association for Cancer Research, cosponsored by the American Society of Preventive Oncology 2004, 13(11 Pt 1):1765-1771.

31. Lange EM, Sarma AV, Ray A, Wang Y, Ho LA, Anderson SA, Cunningham JM, Cooney KA: The androgen receptor CAG and GGN repeat polymorphisms and prostate cancer susceptibility in African-American men: results from the Flint Men's Health Study. Journal of human genetics 2008, 53(3):220-226.

32. Esteban E, Rodon N, Via M, Gonzalez-Perez E, Santamaria J, Dugoujon JM, Chennawi FE, Melhaoui M, Cherkaoui M, Vona G et al: Androgen receptor CAG and GGC polymorphisms in Mediterraneans: repeat dynamics and population relationships. Journal of human genetics 2006, 51(2):129-136.

33. Kittles RA, Young D, Weinrich S, Hudson J, Argyropoulos G, Ukoli F, Adams-Campbell L, Dunston GM: Extent of linkage disequilibrium between the androgen receptor gene CAG and GGC repeats in human populations: implications for prostate cancer risk. Human genetics 2001, 109(3):253-261.

34. Gaston KE, Kim D, Singh S, Ford OH, 3rd, Mohler JL: Racial differences in androgen receptor protein expression in men with clinically localized prostate cancer. The Journal of urology 2003, 170(3):990-993.

35. Olapade-Olaopa EO, Muronda CA, MacKay EH, Danso AP, Sandhu DP, Terry TR, Habib FK: Androgen receptor protein expression in prostatic tissues in Black and Caucasian men. The Prostate 2004, 59(4):460-468.

36. Watson PA, Chen YF, Balbas MD, Wongvipat J, Socci ND, Viale A, Kim K, Sawyers CL: Constitutively active androgen receptor splice variants expressed in castration-resistant prostate cancer require full-length androgen receptor. Proceedings of the National Academy of Sciences of the United States of America 2010, 107(39):16759-16765.

37. Guo Z, Yang X, Sun F, Jiang R, Linn DE, Chen H, Kong X, Melamed J, Tepper CG, Kung HJ et al: A novel androgen receptor splice variant is up-regulated during prostate cancer progression and promotes androgen depletion-resistant growth. Cancer research 2009, 69(6):2305-2313.

38. Chan SC, Li Y, Dehm SM: Androgen receptor splice variants activate androgen receptor target genes and support aberrant prostate cancer cell growth independent of canonical androgen receptor nuclear localization signal. The Journal of biological chemistry 2012, 287(23):19736-19749.

39. Li Y, Chan SC, Brand LJ, Hwang TH, Silverstein KA, Dehm SM: Androgen receptor splice variants mediate enzalutamide resistance in castration-resistant prostate cancer cell lines. Cancer research 2013, 73(2):483-489.

40. de Bono, J.S., et al., Circulating tumor cells predict survival benefit from treatment in metastatic castration-resistant prostate cancer. Clin Cancer Res, 2008. 14(19): p. 6302-9.

41. Rubinstein L., E. Korn, B. Freidlin, et al. Design issues of randomized Phase II trials and a proposal for Phase II screening trials. J. of Clinical Oncology, 23(28):7199-7206, 2005.

42. Fleiss, J.L., Tytun, A., Ury, S.H.K. A simple approximation for calculating sample sizes for comparing independent proportions. Biometrics, 36:343-346, 1980.

14.0 Data and Safety Monitoring

Scheduled meetings/ teleconferences will be held monthly or more frequently depending on the activity of the protocol. These meetings will include the protocol investigators and data managers involved with the conduct of the protocol. The Data and Safety Monitoring Committee at the coordinating site (Karmanos Cancer Institute) will have oversight of all patients enrolled on the trial at all participating sites.

14.1 During these meetings the investigators will discuss matters related to:

- Safety of protocol participants (Adverse Event reporting)
- Validity and integrity of the data
- Enrollment rate relative to expectation of target accrual, characteristics of participants
- Retention of participants, adherence to the protocol (potential or real protocol violations)
- Data completeness on case report forms and complete source documentation

14.2 Completed Data and Safety Monitoring Reports of these regular investigator meetings will be kept on file in the office of the Clinical Trials Core (see form in appendix C). The data manager assigned to the clinical trial will be responsible for completing the report form. The completed reports will be reviewed and signed off by the Principal Investigator (PI) or by one of the Co-investigators in the absence of the PI. The signed off forms will then be forwarded to the Director, Clinical Trials Core for review of completeness and processing with the Data and Safety Monitoring Committee.

14.4 The Barbara Ann KARMANOS Cancer Institute, Data and Safety Monitoring Committee will meet on a regular basis to review the prior Serious Adverse Event forms and Data and Safety Monitoring study specific reports that have been filed.

15.0 APPENDICES

APPENDIX A: Guidance on Reducing the Risks of Pharmacokinetic Drug-Drug Interactions with enzalutamide/MDV3100

There is a potential for other medicinal products to affect MDV3100 exposures and for MDV3100 to affect exposures to other medicinal products. Examples of drugs with the potential for drug-drug interactions with MDV3100 are provided below. For the most current list of drugs that may be subject to drug-drug interactions, consult the following sources:

□ http://www.fda.gov/drugs/developmentapprovalprocess/developmentresources/druginteractionslabeling/ucm093664.htm

□ http://medicine.iupui.edu/clinpharm/ddis/table.aspx

Potential for Other Medicinal Products to Affect MDV3100 Exposures

CYP2C8 inhibitors and inducers

Clinical data indicate that CYP2C8 plays an important role in the metabolism of MDV3100; therefore, strong inhibitors (e.g., gemfibrozil) or inducers (e.g., rifampicin) of CYP2C8 should be used with caution during MDV3100 treatment.

Potential for MDV3100 to Affect Exposures to Other Medicinal Products

Enzyme induction

Clinical data indicate that MDV3100 is a strong inducer of CYP3A4 and a moderate inducer of CYP2C9 and CYP2C19. UGT may be induced as well. These results suggest that MDV3100 causes enzyme induction via activation of PXR. Medicinal products with a narrow therapeutic range that are substrates of CYP3A4, CYP2C9, CYP2C19, or UGT should be used with caution when administered concomitantly with MDV3100 and may require dose adjustment to maintain therapeutic plasma concentrations. Such substrates include, but are not limited to:

- Macrolide antibiotics (e.g., clarithromycin)
- Benzodiazepines (e.g., diazepam, midazolam)
- Immune modulators (e.g., cyclosporine, tacrolimus)
- HIV antivirals (e.g., indinavir, ritonavir)
- Anti-epileptics (e.g., phenobarbitone, phenytoin,)
- Coumarins (e.g., warfarin)
- Certain anti-cancer agents (e.g., cabazitaxel, irinotecan, sunitinib)

In consideration of the long half-life of MDV3100 (approximately 1 week), effects on enzymes may persist for 1 month or longer after stopping MDV3100.

P-gp substrates

In vitro data indicate that MDV3100 may be a P-gp inhibitor. The effects of MDV3100 on P-gp substrates have not been evaluated in vivo; however, under conditions of clinical use, MDV3100 may be an inducer of P-gp via activation of PXR. Medicinal products with a narrow therapeutic range that are substrates for P-gp (e.g., colchicine, dabigatran etexilate, digoxin) should be used with caution when administered concomitantly with MDV3100

APPENDIX B

Determination of progression by PSA per PCWG2 criteria

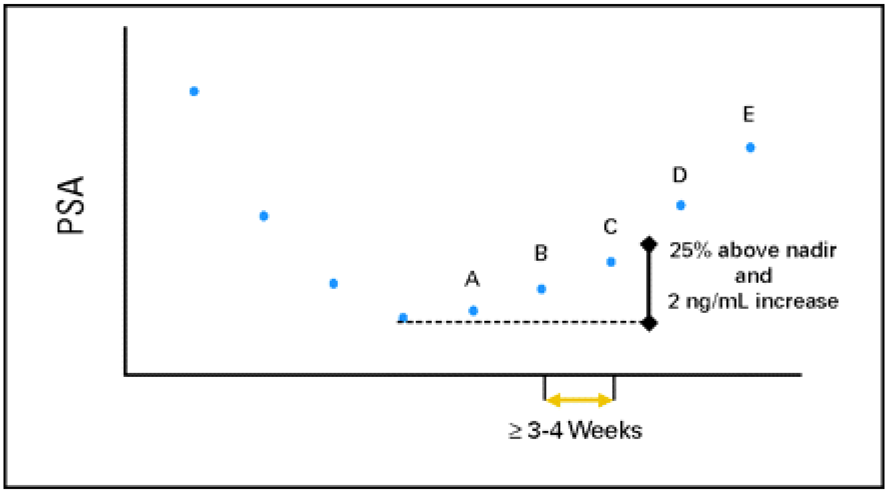

Prostate-specific antigen (PSA) progression. An increase of 25% and absolute increase of 2 ng/mL or more above the nadir. Values A, B, and C show rising PSA values that do not meet the criteria. Value D is the first PSA value that is greater than 25% and more than 2 ng/mL above the nadir, confirmed with a further rise in PSA shown by value E. For reporting purposes, PSA progression would be recorded on the date value D was obtained. (See example above)

APPENDIX C

Barbara Ann KARMANOS Cancer Institute Protocol Specific Data and Safety Monitoring Report

**PROTOCOL #:**		**DATE:**	

**PROTOCOL TITLE**		
ATTENDANCE		

PROTOCOL ACTIVITY SINCE LAST REPORT			
Accrual Goal: List multi-stages individually		Accrual to Date: List multi-stages individually	
Date of Last Report:		Accrual Since Last Report:	
Eligible:		Ineligible (provide reason):	

Specifically for Phase I Trial &/or Dose Escalating Trials:

**Dose Level**	**Accrual**
1	
2	
3	

SAE REPORTING List date of occurrence and date IRB notified [attach SAE reports & SAE spreadsheet].
1]
2]
**OFF TREATMENT Provide reason [progression, death, toxicity, completed therapy, etc].**
**PROTOCOL VIOLATIONS Deviations from protocol treatment, monitoring, or study calendar.**
**PROTOCOL AMENDMENTS Include date submitted to regulatory bodies**

**and date approved.**
**OTHER COMMENTS**

**Investigator Signature:**		**Data Manager Signature:**	

2008
