# Supplementary material for: Clinical Efficacy of Enzalutamide vs Bicalutamide Combined With Androgen Deprivation Therapy in Men With Metastatic Hormone-Sensitive Prostate Cancer: A Randomized Clinical Trial
Source: JAMA Netw Open. 2021 Jan 26;4(1):e2034633. doi: 10.1001/jamanetworkopen.2020.34633 (PMC7838941; doi:10.1001/jamanetworkopen.2020.34633)
Supplement: Supplement 2. — eTable. Grade 3 Toxicities by Treatment Arm That Were Clinically Relevant and Deemed to Be Probably or Definitely Related to Therapy (N = 69) eFigure. Standard Curves for Biomarkers [file jamanetwopen-e2034633-s002.pdf]

Supplemental Online Content

Vaishampayan UN, Heilbrun LK, Monk P III, et al. Clinical efficacy of enzalutamide vs bicalutamide combined with androgen deprivation therapy in men with metastatic hormone-sensitive prostate cancer: a randomized clincial trial. *JAMA Netw Open*. 2021;4(1):e2034633. doi:10.1001/jamanetworkopen.2020.34633

eTable. Grade 3 Toxicities by Treatment Arm That Were Clinically Relevant and Deemed to Be Probably or Definitely Related to Therapy (N = 69)

eFigure. Standard Curves for Biomarkers

This supplemental material has been provided by the authors to give readers additional information about their work.

© 2021 Vaishampayan UN et al. *JAMA Network Open.*

eTable. Grade 3 Toxicities by Treatment Arm That Were Clinically Relevant and Deemed to Be Probably or Definitely Related to Therapy (N = 69)

	Treatment Arm (N=69)		
Type of toxicity	Enzalutamide(n=35)	Bicalutamide(n=34)	Total (n=69)
Seizure	1 (3%)	0	1 (1%)
Hypertension	3 (9%)	1 (3%)	4 (6%)
Fatigue	0 (0%)	2 (6%)	2 (3%)
Syncope	2 (6%)	0 (0%)	2 (3%)
Hot flashes	0 (0%)	1 (3%)	1 (1%)
Injection site reaction	0 (0%)	1 (3%)	1 (1%)
Weight gain	1 (3%)	0 (0%)	1 (1%)

Arm A = Ezalutamide plus LHRH analogue; Arm B = Bicalutamide plus LHRH analogue.

Only treated patients were evaluated for toxicity. One patient on each Arm was never treated after randomization.

Only toxicities that were deemed at least possibly related to enzalutamide or bicalutamide were included.

There were no Grade 4 or Grade 5 toxicities that were related to treatment.

Table entries reflect the worst Grade of each type of toxicity.

Table entries are the number of patients who experienced a Grade 3 toxicity of a given type, and (in parentheses) that number as a percentage of treated patients on that Arm, or for both Arms combined.

© 2021 Vaishampayan UN et al. *JAMA Network Open.*

eFigure 1. Standard Curves for Biomarkers

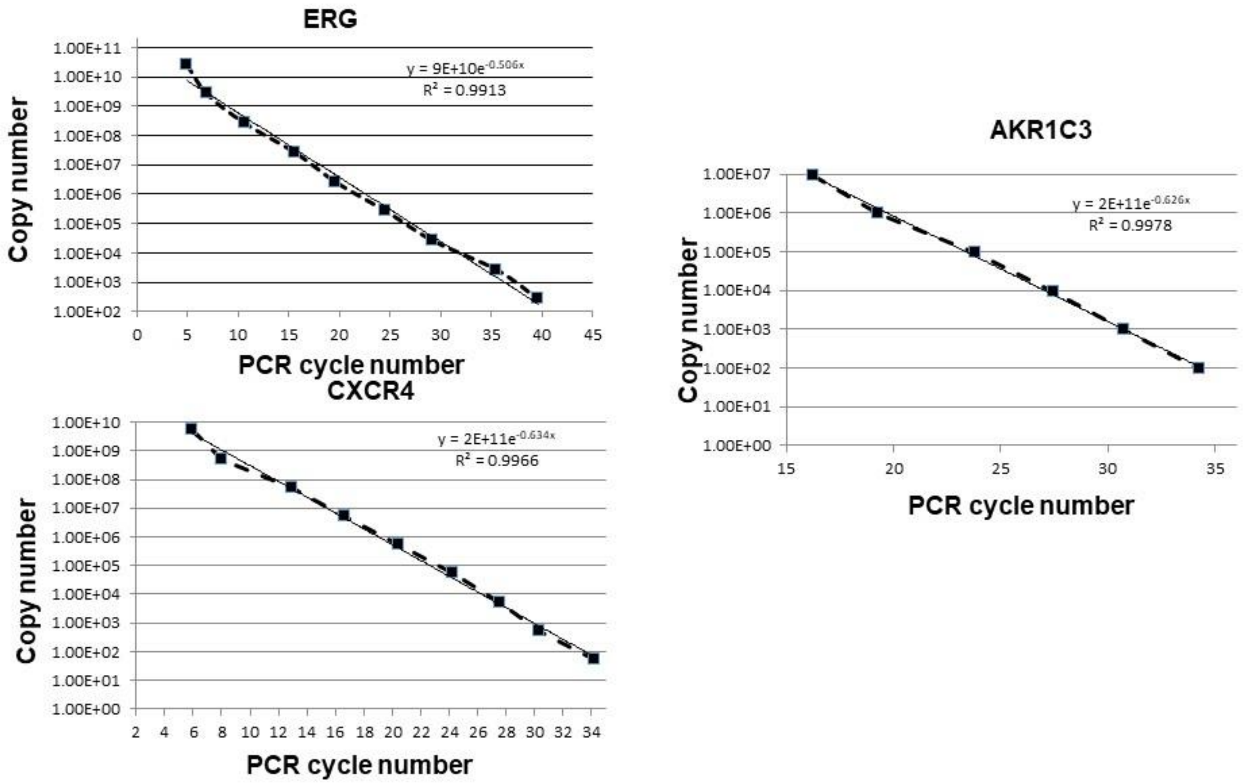

© 2021 Vaishampayan UN et al. *JAMA Network Open.*
