# Supplementary material for: Clinical Efficacy of Enzalutamide vs Bicalutamide Combined With Androgen Deprivation Therapy in Men With Metastatic Hormone-Sensitive Prostate Cancer: A Randomized Clinical Trial
Source: JAMA Netw Open. 2021 Jan 26;4(1):e2034633. doi: 10.1001/jamanetworkopen.2020.34633 (PMC7838941; doi:10.1001/jamanetworkopen.2020.34633)
Supplement: Supplement 3. — Data Sharing Statement [file jamanetwopen-e2034633-s003.pdf]

Data Sharing Statement

Vaishampayan. Clinical Efficacy of Enzalutamide vs Bicalutamide Combined With Androgen Deprivation Therapy in Men With Metastatic Hormone-Sensitive Prostate Cancer. *JAMA Netw Open*. Published January 26, 2021. doi:10.1001/jamanetworkopen.2020.34633

Data
Data available: Yes
Data types: Deidentified participant data
How to access data: Available on review of request
When available: With publication

Supporting Documents
Document types: None

Additional Information
Who can access the data: researchers whose proposed use of the data has been approved
Types of analyses: Meta analysis
Mechanisms of data availability: with investigator support, after approval of a proposal and with a signed data access agreement).
Any additional restrictions: N/A
